# Hyperactivation of mTORC1 in a double hit mutant zebrafish model of tuberous sclerosis complex causes increased seizure susceptibility and neurodevelopmental abnormalities

**DOI:** 10.3389/fcell.2022.952832

**Published:** 2022-09-27

**Authors:** Ann-Sofie De Meulemeester, Lise Heylen, Aleksandra Siekierska, James D. Mills, Alessia Romagnolo, Nicole N. Van Der Wel, Eleonora Aronica, Peter A. M. de Witte

**Affiliations:** ^1^ Laboratory for Molecular Biodiscovery, KU Leuven, Leuven, Belgium; ^2^ Department of (Neuro)Pathology Amsterdam Neuroscience, Amsterdam UMC Location University of Amsterdam, Amsterdam, Netherlands; ^3^ Department of Clinical and Experimental Epilepsy, UCL Queen Square Institute of Neurology, London, United Kingdom; ^4^ Chalfont Centre for Epilepsy, Chalfont St Peter, United Kingdom; ^5^ Department of Medical Biology, Electron Microscopy Center Amsterdam, Amsterdam UMC Location University of Amsterdam, Amsterdam, Netherlands; ^6^ Stichting Epilepsie Instelling Nederland (SEIN), Heemstede, Netherlands

**Keywords:** mTOR, tuberous sclerosis complex (TSC), zebrafish, epilepsy, neurodevelopment, RNA-sequencing, SEGA

## Abstract

Tuberous sclerosis complex (TSC) is a multisystem genetic disorder caused by pathogenic variants in *TSC1* and *TSC2* genes. TSC patients present with seizures and brain abnormalities such as tubers and subependymal giant cells astrocytoma (SEGA). Despite common molecular and clinical features, the severity of the disease varies greatly, even intrafamilially. The second hit hypothesis suggests that an additional, inactivating mutation in the remaining functional allele causes a more severe phenotype and therefore explains the phenotypic variability. Recently, second hit mutations have been detected frequently in mTORopathies. To investigate the pathophysiological effects of second hit mutations, several mouse models have been developed. Here, we opted for a double mutant zebrafish model that carries a LOF mutation both in the *tsc2* and the *depdc5* gene. To the best of our knowledge, this is the first time a second-hit model has been studied in zebrafish. Significantly, the DEP domain-containing protein 5 (*DEPDC5*) gene has an important role in the regulation of mTORC1, and the combination of a germline *TSC2* and somatic *DEPDC5* mutation has been described in a TSC patient with intractable epilepsy. Our *depdc5* ^*−/−*^x *tsc2* ^*−/−*^ double mutant zebrafish line displayed greatly increased levels of mammalian target of rapamycin (mTORC1) activity, augmented seizure susceptibility, and early lethality which could be rescued by rapamycin. Histological analysis of the brain revealed ventricular dilatation in the *tsc2* and double homozygotes. RNA-sequencing showed a linear relation between the number of differentially expressed genes (DEGs) and the degree of mTORC1 hyperactivity. Enrichment analysis of their transcriptomes revealed that many genes associated with neurological developmental processes were downregulated and mitochondrial genes were upregulated. In particular, the transcriptome of human SEGA lesions overlapped strongly with the double homozygous zebrafish larvae. The data highlight the clinical relevance of the *depdc5* ^−/−^ x *tsc2* ^−/−^ double mutant zebrafish larvae that showed a more severe phenotype compared to the single mutants. Finally, analysis of gene-drug interactions identified interesting pharmacological targets for SEGA, underscoring the value of our small zebrafish vertebrate model for future drug discovery efforts.

## Introduction

mTORopathies are defined as a group of neurodevelopmental diseases characterized by hyperactivity of mammalian target of rapamycin complex 1 (mTORC1). The most intensively studied mTORopathy is tuberous sclerosis simplex (TSC), which is caused by inactivating mutations in one of the tumor suppressor genes *TSC1* or *TSC2*. These genes encode hamartin and tuberin, respectively, which together with TBC1D7 form the TSC complex and play a pivotal role in suppression of mTORC1 (Qin et al., 2016; Muhlebner et al., 2019). mTORC1 is involved in many cellular processes and has a direct impact on the proliferation, differentiation and migration of neuronal cells in the central nervous system (Yu and Cui, 2016; Muhlebner et al., 2019). In fact, of all TSC patients, 90% suffer from epilepsy and about two-thirds of them develop drug-resistant epilepsy (DRE). In addition, focal brain malformations including tubers, subependymal glial nodules (SEN) and subependymal giant cell astrocytomas (SEGA) are present in 90%, 80% and 15% of patients, respectively (Henske et al., 2016). Cortical tubers consist of areas of cortical dyslamination and white matter abnormalities. The size and number of tubers can be correlated with the severity of the epilepsy phenotype (Hulshof et al., 2022). SENs are typically located around the wall of the lateral ventricles and considered as the precursor lesion of SEGAs. SEGAs are slow-growing tumours composed of large ganglion-like astrocytes, with a mixed glio-neuronal feature, and they correspond histologically to astrocytomas WHO grade 1 (Louis et al., 2021). Though being histologically benign, SEGAs can lead to serious neurological complications, including acute hydrocephalus. The relevance of hyperactive mTORC1 signaling in TSC, or mTORopathies in general, is further underscored by the clinical use of mTOR inhibitors for inoperable SEGA lesions and as an add-on treatment of DRE in TSC (Curatolo et al., 2018; Muhlebner et al., 2019; Moavero et al., 2021).

The clinical phenotype of TSC and mTORopathies varies greatly within the patient group, even intrafamilially (Curatolo et al., 2015b). This pleiotropic phenotype can be partly explained by the second hit hypothesis suggesting that the addition of a second somatic mutation to a primary mutation causes an exacerbation of the phenotype. Evidence supporting the second hit hypothesis has been found in a series of mTORopathies (Baldassari et al., 2019; Crino et al., 2010; D’gama et al., 2017; Martin et al., 2017; Ribierre et al., 2018; Sim et al., 2019). Specifically for TSC, second hit mutations have been confirmed especially in SEN/SEGA lesions (Bongaarts et al., 2017; Martin et al., 2017). However, the availability of more sensitive sequencing technologies in recent years has made it possible to detect second hit mutations in tubers as well. For instance, Martin et al. (2017) identified a second hit mutation in 35% of tuber samples tested by targeted deep sequencing.

To investigate the pathophysiological relevance of second hit mutations in brain lesions of TSC patients, many mouse models have been developed (Switon et al., 2017). The first global knockout (KO) mouse models suffered from early embryonic lethality that hampered the behavioral or electrophysiological investigations. Therefore, conditional KO and *in utero* electroporation (IUE) models were developed. These biallelic mutant models display frequently mTORC1 hyperactivity, a disorganized cortex due to the presence of cytomegalic and ectopic neurons and neuronal hyperexcitability, similarly to human TSC patients. Albeit, the absence of a clinically relevant disease phenotype in the monoallelic mice complicates the interpretation of the pathophysiological effects of the second hit mutants. Recently, *in vitro* models using iPSCs from TSC patients have been derived allowing comparison between the effects attributable to mono- and biallelic loss of *TSC1/2* (Costa et al., 2016; Sundberg et al., 2018; Zucco et al., 2018; Winden et al., 2019). Interestingly, the study of Winden *et al.* (Winden et al.) showed higher mTORC1 activity and a stronger phenotype in biallelic mutant iPSC derived from TSC patients compared to the monoallelic ones. Nevertheless, the need for differentiation protocols (Winden et al., 2019) and the absence of different cell types *in vitro* models often fail to properly reproduce the *in vivo* context present in a whole organism.

Over the years, zebrafish models emerged in biomedical research as a powerful tool to investigate neurological diseases. In general, their small size, which facilitates maintenance and experimental procedures, together with their vertebrate background and thus relatively high genetic and physiologic similarity to humans, may explain their increasing popularity. Their transparency and *ex utero* development (Rosch et al., 2019) facilitates the study of neurological developmental processes and epileptiform phenotypes compared to mouse models where *in utero* development in combination with an early lethal phenotype complicates detailed phenotypical investigations. In addition, zebrafish models have also gained interest as drug discovery platform as they combine the strengths of high-throughput drug screening with *in vivo* testing benefits (Macrae and Peterson, 2015; Stewart et al., 2015).

Importantly, heterozygous *tsc2* ^+/−^ mutant zebrafish with a monoallelic deletion of the *tsc2* gene do not have significantly altered levels of mTORC1 activity and gene expression (Scheldeman et al., 2017), and do not show any disease phenotype (Kim et al., 2011; Scheldeman et al., 2017; Kedra et al., 2020). Thus, TSC disease is recessive in zebrafish, while showing an autosomal dominant inheritance pattern in humans. Conversely, biallelic *tsc2* ^−/−^ mutant larvae display a TSC-like phenotype with increased mTORC1 activity, brain abnormalities and spontaneous epileptiform events, recapitulating the pathophysiological effects of a first hit mutation in humans (Kim et al., 2011; Scheldeman et al., 2017; Kedra et al., 2020).

To investigate the pathophysiological effects of a second hit mutation resulting in a further increased mTORC1 activity, we generated a double mutant model carrying a LOF mutation in the *depdc5* gene in the *tsc2* ^*−/−*^ background. We selected the DEP domain-containing protein 5 (*DEPDC5*) gene for its involvement in the regulation of mTORC1. Unlike the TSC complex that functions in response to growth factors and cellular stress levels, DEPDC5 is part of the GATOR1 complex and senses cytosolic amino acid levels. In case of amino acid depletion, the GATOR1 complex limits the translocation of mTORC1s to the lysosome and in this way abolishes its subsequent activation (Shimobayashi and Hall, 2016). Accordingly, the mutations present in our *depdc5* ^*−/−*^ *x tsc2* ^*−/−*^ double mutants, which affect both the growth factor- and amino acid and sensing arm, may result in a further increase of mTORC1s activity. Moreover, patients with *DEPDC5* mutations present an epilepsy phenotype, with focal familial epilepsy with variable foci (FFEVF) being the most frequent, and in case of second hit mutations, they show brain abnormalities such as focal cortical dysplasia (FCD) type II. The somewhat comparable disease phenotype to TSC (Scheffer et al., 2014) and the identification of second hit mutations in this gene (Anderson, 2018), suggest a similar pathophysiological mechanism caused by *DEPDC5* and *TSC2* loss. Finally, from a practical point of view, a *depdc5* LOF zebrafish model has been extensively characterized for morphological and functional brain alterations, and displayed a strong mTORC1-dependence (Swaminathan et al., 2018).

Taken together, the *depdc5* ^*−/−*^x *tsc2* ^*−/−*^ double mutant zebrafish larvae showed a more severe phenotype compared to the single mutants, including neurodevelopmental abnormalities and an increased susceptibility to seizures. In addition, we demonstrated a linear relationship between the degree of mTORC1 hyperactivity and the number of differentially expressed genes (DEGs) in the different mutant zebrafish larvae used. Overall, our phenotypic and transcriptomic study shows that the second-hit model in zebrafish recapitulates many features of the human disease and may be useful for identifying new treatment options for SEGA.

## Material and methods

### Zebrafish husbandry

Zebrafish (*Danio rerio*) adults were maintained at 28 ± 2°C on a 14:10 h light/dark cycle under standard aquaculture conditions. Embryos were collected via natural spawning and immediately transferred to embryo medium, containing 1.5 mM HEPES buffer (pH 7.2), 17.4 mM NaCl, 0.21 mM KCl, 0.18 mM Ca(NO_3_)_2_, 0.12 mM MgSO_4,_ and 0.6 µM methylene blue. For all experiments, larvae at 0–10 days post fertilization (dpf) were used and kept in an incubator on a 14 h light/10 h dark cycle at 28.5°C. All zebrafish experiments were approved by the Ethical Committee’s regulations of the University of Leuven (project number P165/2020, approval number LA1210261).

### Zebrafish strains

Heterozygous zebrafish carrying the *depdc5* ^*udm102/+*^ mutation (gift from Dr. E. Samarut, University of Montreal) were outcrossed with heterozygous zebrafish carrying the *tsc2* ^*vu242/+*^ mutation (gift from Dr. K. Ess, Vanderbilt University). The offspring was raised to adulthood and genotyped for both mutations by high resolution melting (HRM) technique. Subsequently, (*depdc5* ^*+/−*^ *x tsc2* ^+/−^) double heterozygotes (F1 generation) were selected and in-crossed. The wild-type, *depdc5* ^*−/−*^ *, tsc2* ^*−/−*^ *, and depdc5* ^*−/−*^ *x tsc2* ^−/−^ F2 generation larvae were produced in a Mendelian ratio of 1:16 (Supplementary Figure S1A), identified by HRM, and used for the experiments.

*Tg[dlx5a/dlx6a-EGFP]* (Noble et al., 2015) *x Tg[vglut2a:loxP-RFP-loxP-GFP]* (Satou et al., 2012) transgenic line was crossed with (*depdc5* ^*+/−*^ *x tsc2* ^+/−^) double heterozygous adults. The F1 generation larvae were screened for the presence of EGFP and RFP fluorescence and later genotyped to identify double heterozygotes amongst the fluorescent transgenic adults. For the experiments, double heterozygote transgenic adults were mated and the genotypes of interest in the F2 progeny were identified by HRM.

### Genotyping

For genotyping zebrafish larvae or adults were anesthetized in a solution of tricaine methanesulfonate (MS222, 0.765 mmol/L). Depending on the experiment performed, a fin clip, the trunks or whole larvae were used for the extraction of crude genomic DNA. The samples were lysed in 20 µl 50 mM NaOH by heating them for 10 min at 100°C. Afterwards, the reaction was neutralized by the addition of 2 µl of 100 mM TrisHCl (pH 8). For genotyping, 2 μl of genomic DNA was mixed with 5 μL of the Precision Melt Supermix for HRM analysis (Bio-Rad #172–5112), 0.5 μl of each primer (10 μM) and 2 µl milliQ water. The following primers were used; ATGCGCTGTTTGGTGAGG (*depdc5*, forward primer), TCC​AGG​AGT​GGG​TGT​TTT​TG (*depdc5*, reverse primer), GAG​ACC​TGC​CTG​GAC​ATG​AT (*tsc2*, forward primer) and CTT​GGG​CAG​AGC​AGA​GAA​GT (*tsc2*, reverse primer). The HRM reaction was performed in the CFX96 touch RT-qPCR detection system (BioRad, RRID:SCR_018064) using CFX Maestro^TM^ Software (Biorad). The melt peaks were automatically clustered by Precision Melt Analysis^TM^ Software (Biorad).

### Survival assay

At 3 dpf zebrafish embryos were arrayed individually in a 96-cell plate in 100 µl embryo medium per well. Survival was followed daily from 3 until 10 dpf and general morphology, heartbeat, and touch response were recorded. The effect of 10 µM rapamycin (Cayman Chemical) was assessed by treating the larvae from 4 dpf onwards with the compound by immersion. The rapamycin solution was replenished daily.

### Morphological measurements

5 dpf larvae were immobilized in 3% methylcellulose and the images were taken using a Leica MZ 10F microscope with a Leica DFC310 FX digital colour camera and Leica Application Suite V3.6 software. The jaw, abdominal, eye, and various head parameters were measured in a blinded way using ImageJ software (RRID: SCR_003070).

### Western blotting

For western blot sampling, wild-type, *depdc5* ^*−/−*^, *tsc2* ^*−/−*^, or *depdc5* ^*−/−*^x *tsc2* ^*−/−*^ zebrafish larvae were anesthetized and decapitated at 5 dpf. The trunks were used for genotyping by HRM and the heads were further processed for SDS-PAGE and immunoblotting. For each genotype, 8-10 heads per sample were lysed in RIPA buffer (Sigma-Aldrich) supplemented with protease inhibitors (Roche). Subsequently, the BCA assay (Thermo Fisher Scientific) was used to load equal amounts of proteins (20–40 μg) on 4%–12% Bis-Tris gel (Novex, Life Technologies) and the SDS-PAGE was performed with MOPS running buffer (Novex, Life Technologies). The separated proteins were transferred by a dry transfer (iBlot dry blotting system, Thermo Fisher Scientific) to a nitrocellulose membrane, which was blocked for 1 h at RT in the Intercept Blocking Buffer (Li-Cor) and then incubated overnight at 4°C with a primary antibody against pS6 (1:1000 dilution, Cell Signaling Technology Cat# 2211, RRID:AB_331679) or against phospho-4E-bp1 (1:1000 dilution, Cell Signaling Technology Cat# 2855, RRID: AB_560835) in Intercept Blocking Buffer. The next day, the membrane was washed, incubated with Dylight secondary goat antibody (1:10,000 dilution, (Thermo Fisher Scientific Cat# SA5-35571, RRID:AB_2556775) in 1xTBST for 1 h at RT, and the proteins were visualized with the ChemiDoc Imaging System (Bio-Rad). As a loading control, a rabbit antibody against GAPDH (1:1000 dilution, Sigma-Aldrich, Cat# SAB2701825) was used. The proteins were semi-quantified using Image Studio Lite ver 5.2 (RRID:SCR_013715).

### SUnSET assay

4 dpf larvae were incubated in embryo medium containing 200 μg/ml puromycin (Sigma-Aldrich) (Hornberg et al., 2016). The next day, they were decapitated, and for each genotype, 8-10 heads were pooled per sample and homogenized in RIPA buffer (Sigma-Aldrich) supplemented with protease inhibitors (Roche). Subsequently, equal amounts of proteins (40 μg) were loaded on 4%–12% Bis-Tris gel (Novex, Life Technologies) and the SDS-PAGE was performed with MOPS running buffer (Novex, Life Technologies). The separated proteins were transferred by a dry transfer (iBlot dry blotting system, Thermo Fisher Scientific) to a nitrocellulose membrane, which was blocked for 1 h at RT in the Intercept Blocking Buffer (Li-Cor) and then incubated overnight at 4°C with a primary antibody against puromycin (1:1000 dilution, Millipore, Cat#MABE343, RRID:AB_2566826) in Intercept Blocking Buffer. The next day, the membrane was washed, incubated with the goat anti-mouse secondary antibody (1:10,000 dilution, (Thermo Fisher Scientific Cat# SA5-35571, RRID:AB_2556775) in 1xTBST for 1 h at RT, and the proteins were visualized with the Amersham^TM^ Typhoon^TM^ NIR imager. As a loading control, a rabbit antibody against GAPDH (1:1000 dilution, Sigma-Aldrich, Cat# SAB2701825) was used. The proteins were semi-quantified using Image Studio Lite ver 5.2 (RRID:SCR_013715).

### Confocal imaging

*Tg[dlx5a/dlx6a-EGFP] x Tg[vglut2a:loxP-RFP-loxP-GFP]* (Satou et al., 2012; Noble et al., 2015) zebrafish larvae with biallelic loss of the *depdc5* ^*−/−*^, *tsc2* ^*−/−*^, or *depdc5* ^*−/−*^ x *tsc2* ^*−/−*^ genes were anesthetized at 5 dpf with 0.765 mmol/L MS222, immobilized in 2% low melting point agarose, and positioned on a cover glass for imaging purpose. A two-photon LSM 780 confocal microscope (Zeiss) equipped with an LD LCI Plan Apo 25x/0.8 objective was used to visualize glutamatergic and GABAergic fluorescent neurons in the optic tectum. EGFP and RFP markers were excited at 488 and 561 nm, and emission was recorded at 493/548 nm and 593/656 nm, respectively. Analysis of the stacks was performed with Imaris 9.6.1 software (RRID:SCR_00737). The software detected fluorescent objects with a sphere diameter of 3.5 μm and automatically calculated area and volume parameters.

### Histological procedures

Wild-type, *depdc5* ^*−/−*^ *, tsc2* ^*−/−*^, and *depdc5* ^*−/−*^ *x tsc2* ^*−/−*^ larvae (5 dpf) were anesthetized in 0.765 mmol/L MS222, fin clipped for genotyping and euthanized with an overdose of MS222. Immediately afterwards, the larvae were fixed in 4% of PFA overnight at 4°C and the next day the fixative was replaced with 70% ethanol. Subsequently, at least four larvae per the genotype were embedded in 1% agarose in 1x TAE buffer blocks. The blocks were gradually dehydrated in an enclosed automated tissue processor (Shandon Excelsior ES, Thermo Scientific) and subsequently embedded in paraffin. The embedded larvae were sectioned on a HM 325 manual rotary microtome (Thermo Fisher Scientific) at a thickness of 5 μm. The specimens were stained with haematoxylin and eosin (H&E stain) using Varistain™ Gemini ES Automated Slide Stainer (Thermo Fisher Scientific) according to laboratory protocols. The resulting sections were imaged (20x and 40x) by SPOT 5.1 software (SPOT Imaging, RRID:SCR_014313) using a SPOT-RT3 camera mounted on a Leica microscope.

### Locomotor tracking

Locomotor activity of 5 dpf larvae was investigated using an automated tracking device (Zebrabox^TM^, Viewpoint, Lyon). Individual larvae were arrayed in a 24-well plate using 400 µl embryo medium per well. The protocol consisted of acclimatization for 5 min followed by one cycle of 10 min of light and 10 min of dark. Subsequently, the average total locomotor activity was quantified using the ZebraLab^TM^ software (Viewpoint, Lyon) and expressed in “actinteg” units. Actinteg is defined as the sum of all image pixel changes detected during the time of the tracking experiment. For the PTZ experiments, the 5 dpf larvae were arrayed in a 96-well plate containing 50 μL of embryo medium per well. Subsequently, an equal volume of 10 mM PTZ (Sigma-Aldrich) was added to obtain a final concentration of 5 mM PTZ. The average total locomotor activity was quantified during a 30 min tracking period in the dark using the ZebraLabTM software (Viewpoint, Lyon) and expressed in actinteg units.

### Non-invasive local field potential recordings

Direct measurements of the epileptiform brain activity of 5 dpf wild-type, *depdc5* ^*−/−*^, *tsc2* ^*−/−*^ *,* and *depdc5* ^*−/−*^ x *tsc2* ^*−/−*^ zebrafish larvae were performed by non-invasive LFP recordings. Larvae were immobilized in 2% low melting point agarose (Invitrogen) and a glass pipet (recording electrode) filled with artificial cerebrospinal fluid (124 mM NaCl, 2 mM KCl, 2 mM MgSO_4_, 2 mM CaCl_2_, 1.25 mM KH_2_PO_4_, 26 mM NaHCO_3_, and 10 mM glucose) was positioned on the skin above the optic tectum of the larvae using a stereomicroscope SZX7 (Olympus). The differential signal between the recording electrode and the reference electrode was amplified 10,000 times by DAGAN 2400 amplifier (DAGAN corporation), band pass filtered at 0.3–300 Hz, and digitized at 2 kHz via a PCI-6251 interface (National Instruments) using WinEDR (John Dempster, University of Strathclyde, United Kingdom) software. Analysis of the 10 min recordings was performed in an automated fashion by power spectral density (PSD) analysis using MatLab R2018 (MATrix LABoratory, USA) software (Li et al., 2020). The resulting PSD estimates were normalized against the wild-type larvae and the data were plotted as mean (±SEM) PSD per larvae over the 0–120 Hz region. Outliers were identified via the iterative Rout test (Q = 1%). For the PTZ experiments, the 5 dpf larvae were arrayed in a 96-well plate containing 100 μl of embryo medium. Subsequently, an equal volume of 10 mM PTZ (Sigma-Aldrich) was added for 15 min before recording to obtain a final concentration of 5 mM PTZ. Electrophysiological recordings were performed and analyzed as described above.

### RNA extractions

RNA extractions were performed on wild-type, *depdc5* ^*−/−*^, *tsc2* ^*−/−*^, and *depdc5* ^*−/−*^ x *tsc2* ^*−/−*^ zebrafish heads (7-10 heads pooled per genotype 5 dpf). RNA was extracted at 4°C using TRIzol (Thermo Fisher Scientific), followed by phenol-chlorophorm extraction, isopropanol precipitation, and two ethanol washes. Next, the RNA pellet was air-dried and dissolved in nuclease-free water (Thermo Fisher Scientific). The concentration and purity of the purified total RNA was determined spectrophotometrically using the Nanodrop ND-1000 (Implen) and the samples were stored at −80°C.

### RNA-sequencing

Wild-type, *depdc5* ^*−/−*^, *tsc2* ^*−/−*^ *,* and *depdc5* ^*−/−*^ x *tsc2* ^*−/−*^ zebrafish larvae (5 dpf) were anesthetized by immersion in 0.765 mmol/L MS222 and decapitated with a razor blade. 10 heads were pooled per genotype in quadruplicate and RNA extractions were performed as described above. Nanodrop ND-1000 (Nanodrop Technologies) was used to determine RNA concentration and purity and Bioanalyser 2100 (Agilent Technologies) was used to assess RNA integrity. 500 ng of total RNA per sample was used as input for sample preparation. Library preparation and sequencing were completed at Macrogen (Europe). Illumina TruSeq Stranded mRNA-Seq sample preparation kit was used to prepare the mRNA sequencing library according to the manufacturer guidelines. This library was subjected to paired-end sequencing using the Novaseq6000. Sequencing data are available in the ArrayExpress database (http://www.ebi.ac.uk/arrayexpress) under accession number E-MTAB-11776.

### Bioinformatic analysis

Read quality was assessed using FastQC v0.11.8 software. Low quality reads were filtered out using Trimmomatic v0.38 (Bolger et al., 2014): low quality leading and trailing bases were removed, and the quality of the body of the reads was assessed with a trimming sliding window of 4 and a Phred score threshold of 20 bases. Reads shorter than 75 nucleotides, reads with no associated forward or reverse read, and reads that aligned to phix illumina were excluded. Reads that passed quality control were aligned to the zebrafish reference genome, GRCz11, using STAR (Dobin et al., 2013). The number of reads that align to each gene in accordance to the Ensembl GRCz11 zebrafish reference annotation were calculated using featureCounts (Liao et al., 2014) from the Subread package. Differential expression analysis was carried out using the R package DESeq2 (Love et al., 2014). Gene expression changes with a Benjamini-Hochberg adjusted *p*-value < 0.05 were considered statistically significant. For comparing the transcriptomes of the double hit zebrafish mutant and human SEGA lesions (Bongaarts et al., 2020), zebrafish identifiers of genes differentially expressed in the double homozygous larvae were converted to their human orthologs using BioMart (Smedley et al., 2015).

### Gene ontology and pathway enrichment analysis

The Kyoto Encyclopedia of Genes and Genome (KEGG) (Kanehisa and Goto, 2000) for zebrafish and Reactome Pathway Database for human were queried to test DEGs in zebrafish larvae and human SEGA lesions for pathway enrichment, respectively, using the Enrichr (Chen et al., 2013) and ReactomePA package (Yu and He, 2016). The Gene Ontology (GO) knowledgebase (Ashburner et al., 2000) for zebrafish and human were queried to test DEGs in zebrafish larvae and human SEGA lesions for gene ontology enrichment, respectively, using the Enrichr package (Chen et al., 2013). Enriched GO term lists containing the top 10 or top 20 terms amongst cellular component and biological process were processed using the “Enrichment Map” plugin for Cytoscape (http://www.cytoscape.org/) to create a visual representation of the GO enrichment (Isserlin et al., 2014).

### Hypergeometric testing and prediction of potential pharmacological targets for SEGA

Hypergeometric testing of mitochondrial genes present in the Human MitoCarta3.0 dataset (Rath et al., 2021) was performed using the phyper() function in R. For this, zebrafish identifiers of differentially expressed genes in the double homozygotes, *tsc2* ^*−/−*^ and *depdc5* ^*−/−*^ larvae were first converted to their human orthologs using BioMart (Smedley et al., 2015). For the identification of potential pharmacological targets, the overlap between SEGA DEGs and the human orthologs of the double homozygous zebrafish DEGs was imported into the “Drug Gene Interaction Database” (DGIdb) version 4.2.0 (Freshour et al., 2021).

### RT-qPCR analysis

Reverse-transcription was performed using 0.5 μg of total purified RNA with the High-Capacity cDNA Reverse Transcription kit (Applied Biosystems) according to the manufacturer’s protocol. Subsequently, the cDNA was diluted (1:20) and amplified using 5 µl of 2x SsoAdvanced Universal SYBR Green Supermix (Bio-Rad), 2.4 µl of milliQ water, and 0.3 µl of both primers for each RT-qPCR reaction. In each well of Hardshell^®^ Low Profile Thin-wall 96-well skirted PCR plates (Bio-Rad) per sample 8 µl of this mastermix was mixed with 2 µl of diluted cDNA and processed on a CFX96 touch RT-qPCR detection system (Bio-Rad, RRID:SCR_018064) under cycling conditions according to the manufacturer’s protocol. The relative expression levels were quantified using the comparative Cq method (ΔΔCq) with CFX Maestro software (Bio-Rad). The transcripts were normalized against housekeeping gene *atf4a* using specific primers. The list of primers and their sequences are included in Supplementary Table S1.

### Electron microscopy procedure

5 dpf zebrafish were fixed with paraformaldehyde and glutaraldehyde. After sampling of the head these were further post-fixed and stained with 1% osmium tetroxide for 1 h. Subsequently, zebrafish heads were dehydrated in a series of 70, 80, 90, 96 and 100% ethanol. Next, the zebrafish heads were incubated in 1:1 resin-alcohol (LX112) mixture for 2–3 h, followed by 100% resin for 2–3 h. Finally, samples were polymerized at 65°C and sectioned in ultrathin sections (70 nm) using a Leica FC6 ultramicrotome. The sections were placed on copper slot grids and visualized with an FEI/Thermo Fisher Tecnai T12 Transmission Electron Microscope (Thermo Fisher Scientific) using a Xarosa camera with an integrated Radius software.

### Mitochondrial copy number

Total DNA was extracted from individual wild-type, *depdc5* ^*−/−*^, *tsc2* ^*−/−*^, or *depdc5* ^*−/−*^x *tsc2* ^*−/−*^ zebrafish head at 5 dpf with the DNeasy Blood and Tissue Kit (Qiagen) according to the manufacturer’s protocol. Subsequently, the relative mitochondrial copy number was determined by RT-qPCR using the following primers amplifying a mitochondrial fragment of 198 bp; CAA​ACA​CAA​GCC​TCG​CCT​GTT​TAC (forward primer), CAC​TGA​CTT​GAT​GGG​GGA​GAC​AGT (reverse primer) (Hunter et al., 2010) and normalized to the nuclear gene *polg1* using the primers GAG​AGC​GTC​TAT​AAG​GAG​TAC (forward primer) and GAG​CTC​ATC​AGA​AAC​AGG​ACT (reverse primers) (Artuso et al., 2012).

### ATP measurement

The protocol used for ATP measurements was based on the protocol described in (Byrnes et al., 2018). At 5 dpf wild-type, *depdc5* ^*−/−*^, *tsc2* ^*−/−*^, or *depdc5* ^*−/−*^x *tsc2* ^*−/−*^ zebrafish were anesthetized in 0.765 mmol/L MS222 and decapitated. For each sample, 5 zebrafish heads were pooled per genotype, the E3 medium was removed and replaced with 30 µl mitochondrial respiration medium, MiR05 (Oroboros instruments, Bioblast) per head. The samples were then homogenized for 10 s using a Pellet mixer with 1.5 ml disposable pestles (VWR). 50 µL of the obtained homogenates was transferred to a 96-well plate. For each sample, two technical replicates were loaded and at least three biological replicates per condition were included. To each of these wells, 50 µl MiR05 was added. Additionally, 6 negative control wells containing 100 µl MiR05 and a three-point serial dilution of ATP in MiR05 from 1 μM to 10 nM of ATP was prepared as a standard curve. Next, the CellTiter-Glo® Luminescent Cell Viability assay (Promega) was performed in accordance with the manufacturer’s protocol. The 96-well plate was incubated for 30 min at RT. Subsequently, 100 µl of CellGlo reagent per well was added and the plate was placed on an orbital shaker for 2 min. Next, after 10 min incubation at RT, luminescence was recorded with the Synergy^TM^ H1 multi-mode microplate reader (BioTek).

### Statistical analysis

For statistical analyses GraphPad Prism 9 (RRID:SCR_002798) was used. Generally, one-way or two-way ANOVA followed by Bonferroni’s or Sidak’s multiple comparison test was used for the comparison of the means between the four groups. For the comparison of the survival curves the log rank (Mantel-Cox) test was used followed by Bonferroni correction. For RT-qPCR experiments the Kruskal-Wallis test was used followed by Dunn’s correction for multiple testing. Data are presented as mean ± SEM. For the comparison of the overlapping DEGs between SEGAs and *tsc2* homozygotes with the overlapping DEGs between SEGA and double homozygous zebrafish, a chi square test was used.

## Results

### Double homozygous larvae die prematurely and show strong upregulation of mTORC1

**FIGURE 1:**
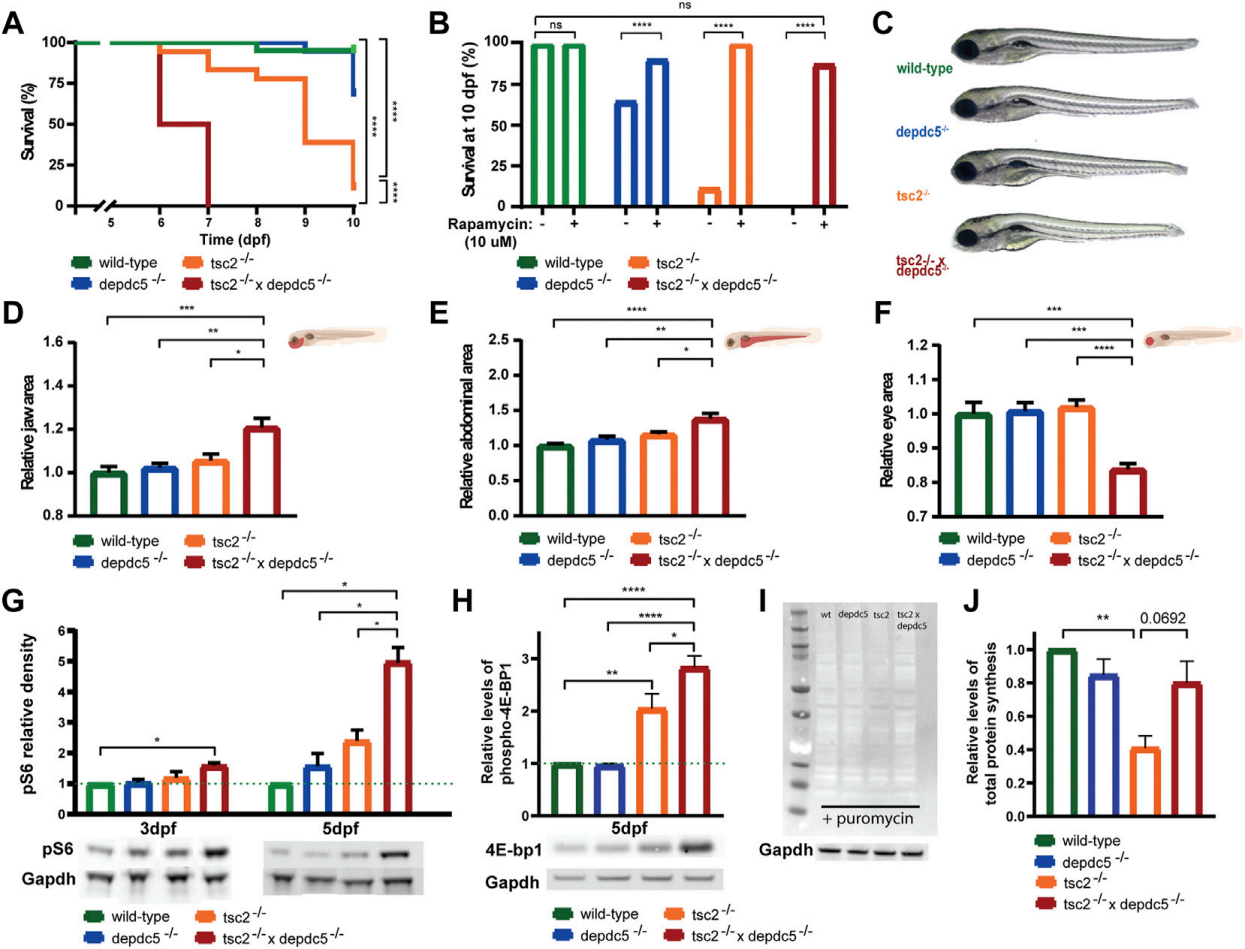
Survival analysis and morphological alterations in double homozygous larvae. **(A)** Kaplan-Meier curves of wild-type, *depdc5* ^−/−^, *tsc2* ^−/−^, and double homozygous larvae. Viability of larvae was assessed daily from 0 dpf until 10 dpf. Significant values (Log rank test) are noted as ****p < 0.0001. **(B)** Survival proportions at 10 dpf of wild-type, *depdc5* ^−/−^, *tsc2* ^−/−^, and double homozygous larvae with or without 10 μM rapamycin treatment. Significant values (one-way ANOVA) are noted as ****p < 0.0001. **(C)** Representative images of 5 dpf wild-type, *depdc5* ^−/−^, *tsc2* ^−/−^, and double homozygous larvae. **(D)–(F)** Quantification of jaw size **(D)**, abdominal area **(E)** and eye size **(F)** in **(C).** Data are presented as mean ± SEM, *n* = 8–10 larvae/condition. Significant values (one-way ANOVA) are noted as ***p ≤ 0.001, **p ≤ 0.01 and *p ≤ 0.05. **(G)** Relative quantification of pS6 protein levels at 3 and 5 dpf in zebrafish heads of wild-type, *depdc5* ^−/−^, *tsc2* ^−/−^, and double homozygous larvae, normalized to Gapdh and represented as fold change expression versus wild-type. Representative blots for pS6 and Gapdh are shown below the graph. Data are presented as mean ± SEM, *n* = 3. Significant values (one-way ANOVA) are noted as ***p ≤ 0.001, **p ≤ 0.01 and *p ≤ 0.05. **(H)** Relative quantification of phospho-4E-bp1 protein levels at 5 dpf in zebrafish heads of wild-type, *depdc5* ^*−/−*^ *, tsc2* ^*−/−*^, and double homozygous larvae, normalized to Gapdh and represented as fold change expression versus wild-type. Representative blots for phospho-4E-bp1 and Gapdh are shown below the graph. Data are presented as mean ± SEM, *n* = 4. Significant values (one-way ANOVA) are noted as ****p ≤ 0.0001, **p ≤ 0.01 and *p ≤ 0.05. **(I)** Representative blot of SUnSET assay following the incorporation of puromycin into newly synthesized proteins in wild-type, *depdc5* ^−/−^, *tsc2* ^−/−^, and double homozygous larvae at 5 dpf. Gapdh was used as loading control and a representative blots for Gapdh is shown below the anti-puromycin blot. **(J)** The average intensity of puromycin signal, normalized to Gapdh signal and represented as fold change expression versus wild-type. Data are represented as ± SEM, *n* = 3. Significant values (one-way ANOVA) are noted as **p ≤ 0.01.

First, we investigated the viability of the wild-type, *depdc5* ^*−/−*^ *, tsc2* ^*−/−*^, and *depdc5* ^*−/−*^ *x tsc2* ^−/−^ larvae (hereafter called *depdc5, tsc2* and double homozygotes, respectively). Between 6 and 7 dpf all double homozygous larvae died, which was significantly earlier than the *depdc5* and *tsc2* homozygotes (Figure 1A, *p* < 0.000001). Importantly, the treatment with the mTOR inhibitor rapamycin could reverse the early lethal phenotype with 87% of double homozygotes surviving until 10 dpf (Figure 1B, *p* > 0.05 compared to wild-type larvae).

From 5 dpf onwards, the double homozygous larvae started to exhibit morphological abnormalities (Figure 1C). Morphologically the double homozygotes displayed a significant increase in jaw (Figure 1D, *p* = 0.0102 compared to *tsc2* homozygotes) and abdomen size (Figure 1E, *p* = 0.0276 compared to *tsc2* homozygotes). On the contrary, eye size was decreased in the double homozygotes (Figure 1F, *p* < 0.0001 compared to *tsc2* homozygotes).

Next, as mTORC1 hyperactivity has shown to be a key feature of TSC *in vitro* models, animal models and resected human tissue (Meikle et al., 2007; Crino et al., 2010; Magri et al., 2011; Magri et al., 2013; Winden et al., 2019), the degree of mTORC1 activity was quantified in zebrafish heads by measuring phospho-S6 (pS6) levels, a well-known downstream substrate of mTORC1, at 3 and 5 dpf. At 3 dpf the double homozygotes already displayed significantly increased pS6 levels as compared to age-matched wild-type larvae (Figure 1G, *p* = 0.0286). Subsequently, elevation of pS6 was also observed in the *depdc5* and *tsc2* homozygotes at 5 dpf. At 5 dpf, the double homozygotes also showed significantly higher pS6 levels compared to the *depdc5* and *tsc2* homozygotes, and wild-types, respectively (Figure 1G, *p* < 0.05). Interestingly, this increase was 4.95-fold compared to that in the wild-type larvae, an outcome that exceeds the sum of the increments seen in the *depdc5* (1.5-fold) and *tsc2* homozygotes (2.4-fold). Accordingly, also the levels of phospho-4E-bp1, another downstream target of mTORC1 (Ben-Sahra and Manning, 2017), were significantly higher in the double homozygous zebrafish larvae compared to the single homozygotes (Figure 1H, *p* ≤ 0.0001 and *p* = 0.0441 compared to the *depdc5* and *tsc2* homozygotes, respectively) and the wild-types (Figure 1H, *p* ≤ 0.0001). Finally, we investigated the difference in total protein synthesis at 5 dpf between the different genotypes using the SUnSET assay. Our results show a significant reduction of total protein synthesis in the tsc2 homozygotes (Figure 1I, *p* = 0.0075), while the level of protein synthesis was somewhat similar in the double homozygotes (Figure 1I, *p* = 0.5532) compared to the wild-type larvae.

### Double homozygous larvae display mild brain alterations

**FIGURE 2:**
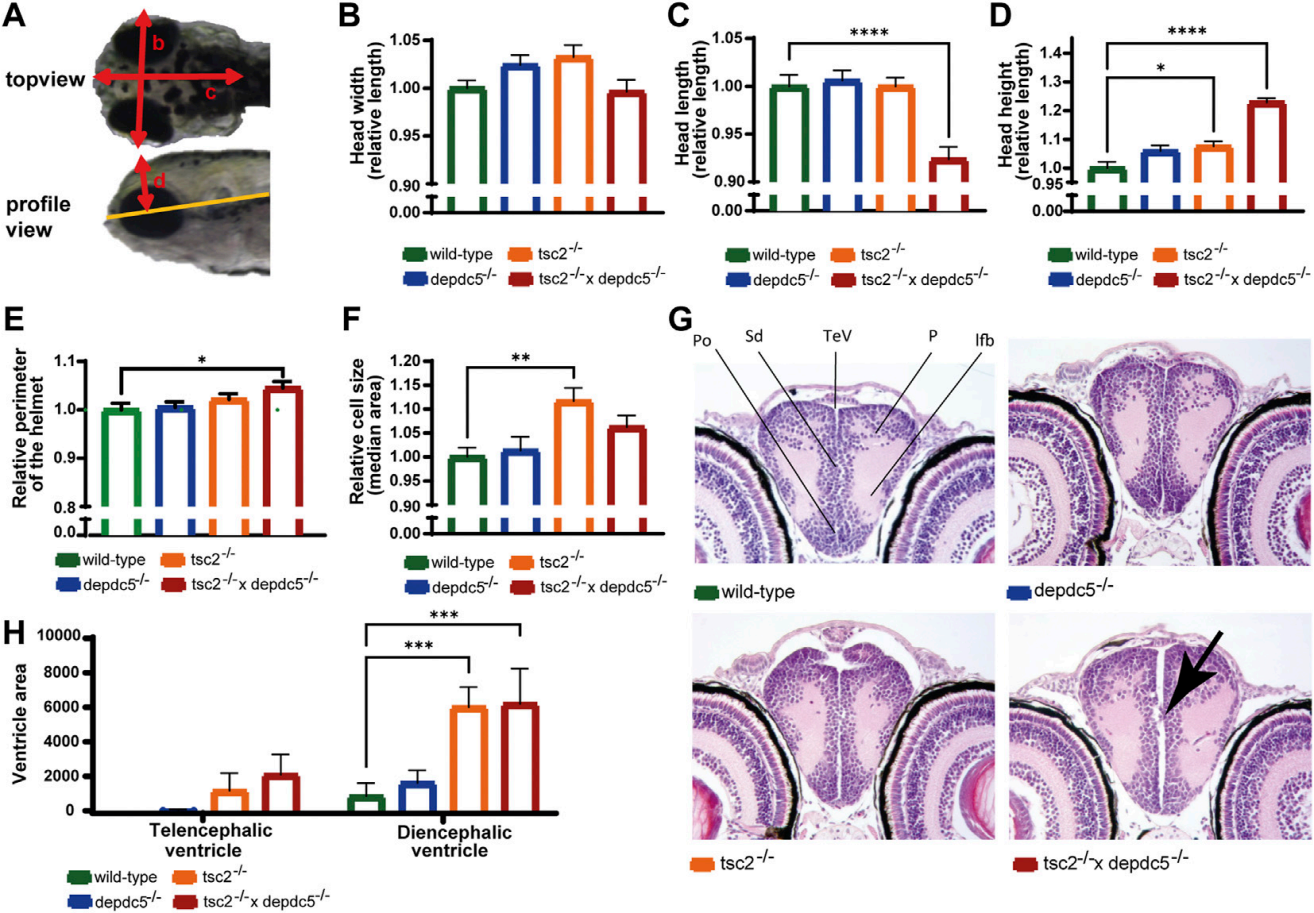
Analysis of the brain abnormalities by microscopy and histology. **(A)** Different head parameters measured in wild-type, depdc^5–/–^, tsc^2–/–^, and double homozygous larvae, with arrows indicating the head width **(B)**, head length **(C)**, head height **(D)** and head helmet **(E)**. **(B–E)** Quantification of the head width **(B)**, head length **(C)**, head height **(D)** and helmet perimeter **(E)** of wild-type, *depdc5* ^−/−^, *tsc2* ^−/−^, and double homozygous larvae at 5 dpf. Absolute lengths were normalized against the wild-type group and relative lengths are reported. Data are presented as mean ± SEM, *n* = 8–11 larvae/condition. Significant values (one-way ANOVA) are noted as****p < 0.0001, *p < 0.05. **(F)** Quantification of the median cell size of EGFP-labelled GABAergic cells in the optic tectum. Data are presented as median ±SEM, n = 12–20 larvae/condition. Significant values (one-way ANOVA) are noted as **p < 0.001. **(G)** Representative 40x images of the anterior diencephalon in wild-type, *depdc5* ^−/−^, *tsc2* ^−/−^ and double homozygous larvae at 5 dpf. *n* = 4 larvae/group. Black arrow indicates the protrusion of cells into the ventricle. **(H)** Quantification of the ventricle area in the pallial and posterior diencephalic area. Data are presented as mean ± SEM, *n* = 4 larvae/condition. Significant values (one-way ANOVA) are noted as ***p < 0.001.

mTORC1 plays a critical role in neurodevelopment and the development of brain malformation as observed in the majority of TSC patients (Curatolo et al., 2015a; Lu et al., 2018), therefore the brain shape and anatomy of the double homozygotes were investigated. As visual inspection at 5 dpf indicated a divergent brain shape in the double homozygotes (Figure 1C), the width, length, and height of the heads were quantified (Figure 2A). The measurements revealed a significant increase in head height but reduced head length in the double homozygotes (Figures 2B–D, *p* > 0.05 for head width, *p* < 0.0001 for head length and *p* < 0.0001 for head height) and suggest that the brain shape has altered. The significant increase of the perimeter of the head helmet (Figure 2E, *p* = 0.0319) illustrates further an enlargement of the brain in the double homozygous larvae.

Subsequently, as enlarged cell size is often observed in *TSC*-deficient models (Meikle et al., 2007; Goto et al., 2011; Magri et al., 2011; Magri et al., 2013; Winden et al., 2019), cell size measurements were performed by confocal imaging of the transgenic line *Tg[dlx5a/dlx6a-EGFP] x Tg[vglut2a:loxP-RFP-loxP-GFP]* homozygous for the *depdc5*, *tsc2* or both genes. Analysis of the fluorescent glutamatergic and GABAergic neurons in the optic tectum revealed a significant increased cell size in the *tsc2* homozygotes as reported in Kim et al. (2011) and a non-significant increase in the double homozygotes (Figure 2F, *p* = 0.0032 and *p* > 0.05, respectively). Furthermore, the amount of glutamatergic and GABAergic cells was not affected in any of the mutants (Supplementary Figures S1B,C, *p* > 0.05).

Histological analysis further showed that all subregions in the forebrain and other major brain regions were present and that gross abnormalities were not present in *tsc2* and double homozygotes (Supplementary Figure S1D). However, the ventricles were enlarged in the *tsc2* and double homozygous larvae, while they were not dilated in the *depdc5* homozygotes (Figure 2G). In particular, the diencephalic ventricles were significantly larger in *tsc2* and double homozygotes than in controls, although the increase of the telencephalic ventricle was non-significant (Figure 2H, *p* > 0.05 for the telencephalic ventricle, *p* < 0.05 and *p* < 0.001 for the diencephalic ventricle in *tsc2* and double homozygotes, respectively). Of interest, two out of four double homozygotes also showed small cellular protrusions invading the ventricle (Figure 2G).

### Double homozygous larvae exhibit increase seizure susceptibility

**FIGURE 3:**
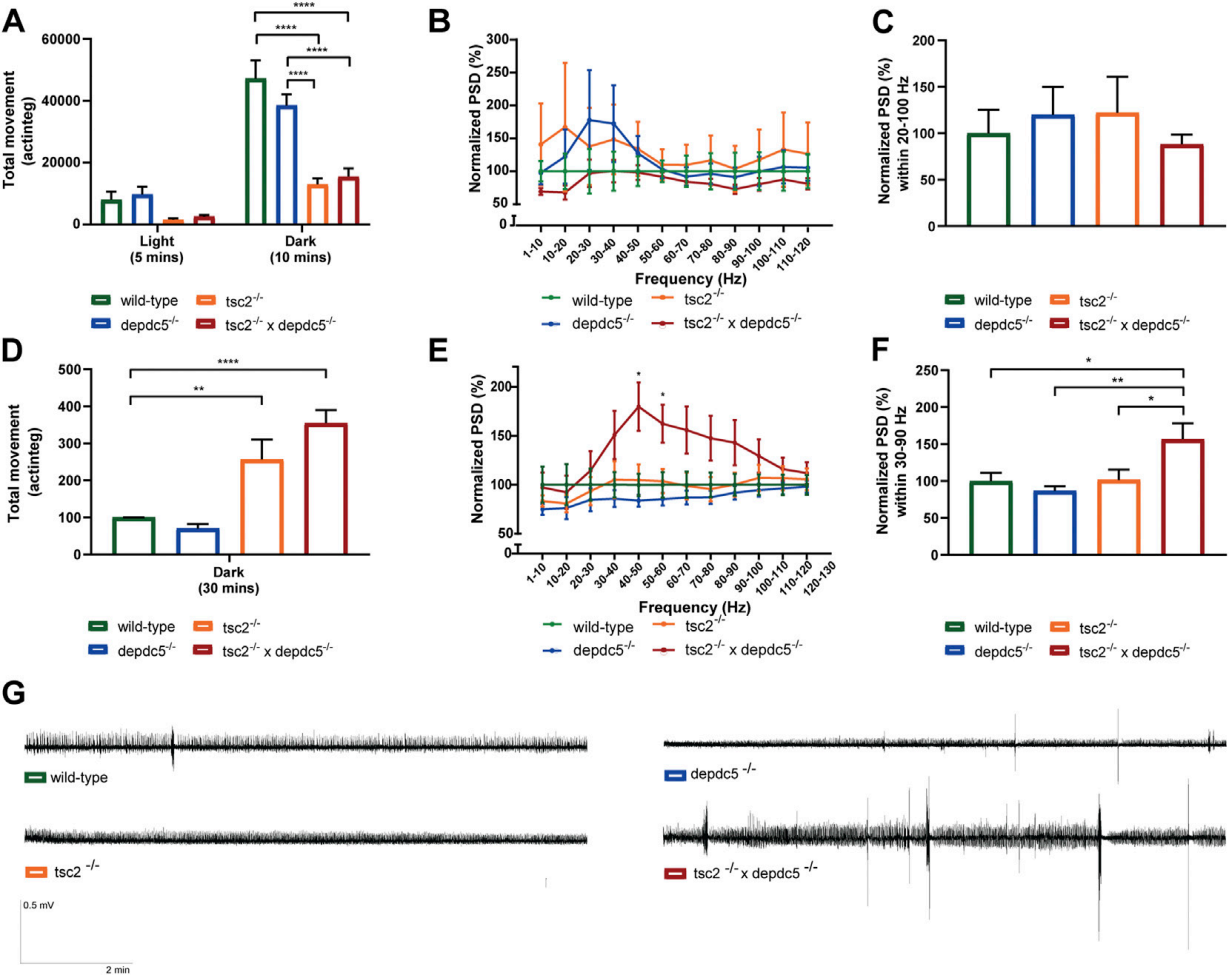
Locomotor behaviour and electrophysiology assessment of double homozygous zebrafish larvae. **(A)** Locomotor activity (average total movement, expressed in actinteg units) tracked during light (5 min) and dark (10 min) periods of wild-type, *depdc5* ^−/−^, *tsc2* ^*−/−*^, and double homozygous larvae at 5 dpf. Data are presented as mean ± SEM, *n* = 13–29 larvae/condition. Significant values (two-way ANOVA) are noted as ****p < 0.0001. **(B,C)** Power spectral density (PSD) ranging from 0 to 120 Hz normalized against the wild-type group. The PSD per 10 Hz **(B)** and per larvae **(C)** over the 20–100 Hz region are plotted as mean ± SEM, *n* = 6–23 larvae/condition. No significant values (one-way ANOVA) were detected, ns *p* > 0.05. **(D)** Locomotor activity tracked in the dark (30 min) in the presence of 5 mM PTZ of wild-type, *depdc5* ^−/−^, *tsc2* ^*−/−*^, and double homozygous larvae at 5 dpf. Data are presented as mean ± SEM, *n* = 8–16 larvae/condition. Significant values (two-way ANOVA) are noted as ****p < 0.0001, **p < 0.01. **(E,F)** Power spectral density (PSD) ranging from 0 to 120 Hz normalized against the wild-type group. All animals were treated with 5 mM PTZ. The PSD per 10 Hz region **(E)** and per larvae over the 30–90 Hz region **(F)** are plotted as mean ± SEM, *n* = 17–26 larvae)/condition. Significant values (one-way ANOVA) are noted as **p < 0.01 and *p < 0.05. **(G)** Representative 10-mins recordings (scale bar; 0.5 mV – 2 min) of wild-type, *depdc5* ^−/−^, *tsc2* ^*−/−*^, and double homozygous larvae at 5 dpf after 5 mM PTZ immersion for 15 min.

Since 80%–90% of TSC patients display epilepsy (Henske et al., 2016), seizure-like behaviour was studied at 5 dpf using a dark-light protocol (5 min light, 10 min dark) to investigate the epilepsy phenotype of the double homozygous larvae. During the dark phase, the double homozygotes displayed significant hypoactivity when compared to the *depdc5* homozygotes and wild-type larvae (Figure 3A, *p* = 0.001), similarly as observed in the *tsc2* homozygotes (Figure 3A, *p* = 0.0150). Next, the epileptiform brain activity was investigated by non-invasive LFP recordings. At 5 dpf the electrographic brain activity was recorded from the optic tectum. Surprisingly, the double homozygotes did not show any increase in spontaneous epileptiform activity compared to the wild-types (Figures 3B,C). Also, the *depdc5* and *tsc2* homozygotes did not display spontaneous epileptiform events at 5 dpf.

Subsequently, seizure susceptibility was investigated by exposing the mutants to a subthreshold dose (5 mM) of pentylenetetrazole (PTZ), a γ-aminobutyric acid type A (GABA_A_) receptor antagonist, commonly used to chemically induce seizures (Afrikanova et al., 2013). In the presence of 5 mM PTZ, the *tsc2* and double homozygotes showed a significant increase in the locomotor activity in comparison to wild-types (Figure 3D, *p* < 0.0001). The double homozygotes moved more when compared to the *tsc2* homozygotes, although this increase was statistically non-significant. Importantly, LFP recordings revealed a significant increase of epileptiform activity in the double homozygotes compared to wild-types (Figures 3E–G, *p* = 0.0277) and *tsc2* homozygotes (Figures 3E–G, *p* = 0.0484 compared to *tsc2* homozygotes). Locomotor and brain activity of *depdc5* homozygotes was not altered by subthreshold doses of PTZ.

### Pathway and GO enrichment analysis of the tsc2 and double homozygotes reveals differential pathological mechanisms

**FIGURE 4:**
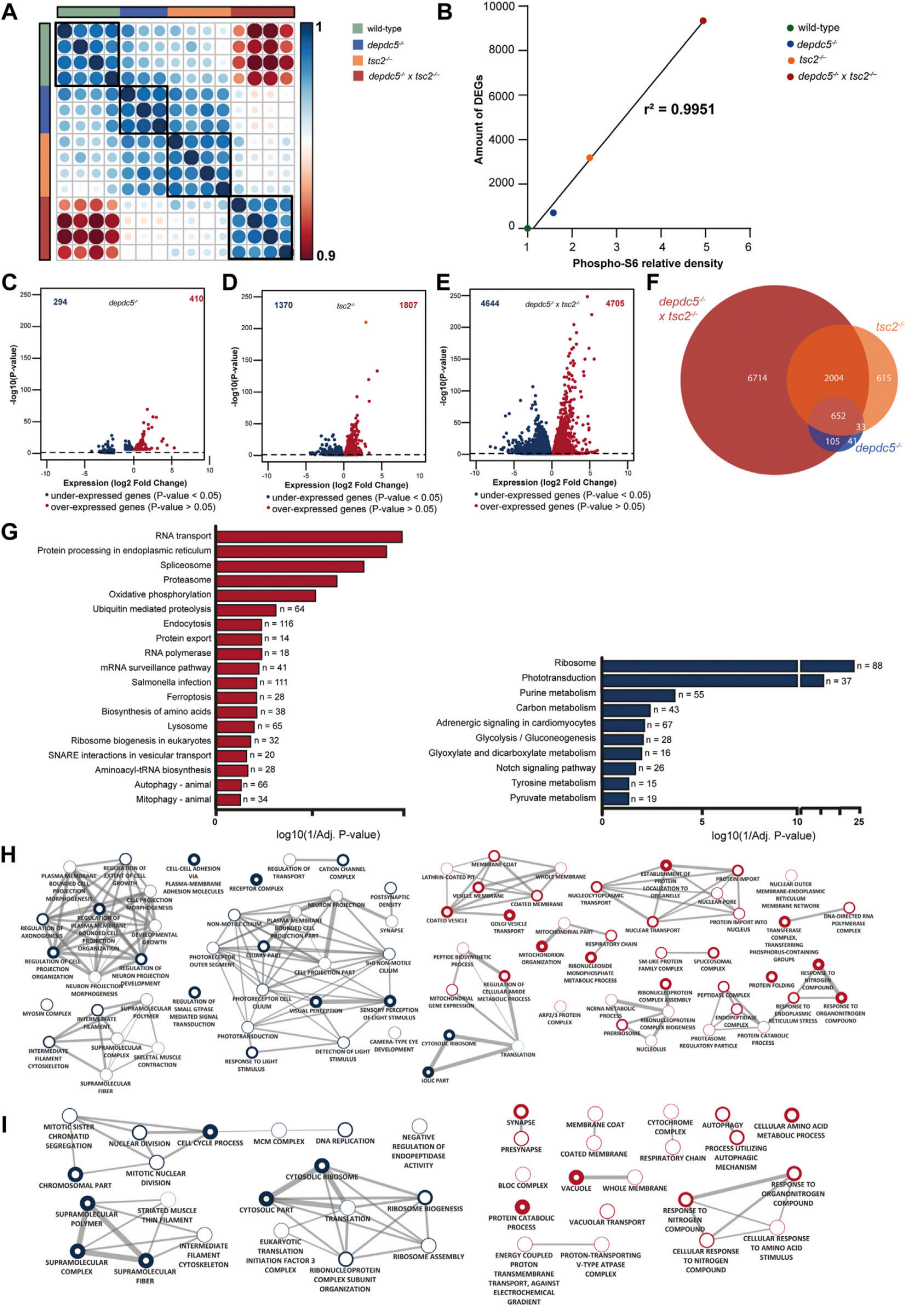
Comparison of the transcriptome profiles of depdc5, tsc2 and double homozygous zebrafish larvae. **(A)** Spearman’s rank correlation matrix of the RNA-Seq data showing separate clustering of wild-types, *depdc5* ^−/−^, *tsc2* ^*−/−*^ *,* and double homozygous larval samples. Areas of the circles show absolute values of corresponding correlation coefficients. Different genotypes are indicated in distinct color codes. Scale bar indicates the strength of the correlation. **(B)** Linear and significant relationship between the levels of pS6 protein and the amount of DEGs. **(C)** Volcano plot showing the differentially expressed genes (DEG) (padj <0.05) between *depdc5* ^−/−^and wild-type larvae. **(D)** Volcano plot showing the DEGs (padj <0.05) between *tsc2* ^*−/−*^ and wild-type larvae. **(E)** Volcano plot showing the DEGs (padj <0.05) between double homozygotes and wild-type larvae. **(F)** Overlap of differentially expressed genes between *depdc5* ^−/−^, *tsc2* ^*−/−*^ *,* and double homozygous zebrafish larvae. For the *depdc5* ^−/−^ and *tsc2* ^−/−^ homozygotes 90% and 80% of the total DEGs, respectively, were found to overlap with that of the double homozygotes. The proportion overlapping DEGs with the double mutant were dependent on the genotype of the zebrafish (Chi-squared test, *p*-value = 3.648e-13). **(G)** Enriched KEGG pathways from differentially expressed genes between double homozygous zebrafish larvae and wild-types. Pathways enriched amongst upregulated genes are indicated in red, pathways enriched amongst downregulated genes are indicated in blue. The x-axis represents the log10 (1/*p*-value), n indicates the number of genes appearing in each pathway. **(H)** GO enrichment map from up and downregulated genes between double homozygous zebrafish larvae and wild-types and **(I)** *tsc2* ^*−/−*^ zebrafish larvae and wild-types. Each node represents a different GO term, the red and blue outside of nodes indicate enrichment in up or downregulated genes, respectively. Green node indicating GO term enriched in both up and downregulated gene lists. The larger the node the greater the number of genes in the enriched GO term. Connecting lines indicate common genes shared between nodes, the thicker the line the more genes in common.

To investigate the transcriptional perturbations underlying the observed phenotypes, RNA-sequencing was performed on the heads of *depdc5, tsc2,* and double homozygous 5 dpf zebrafish larvae. On average, 31 million paired-end reads were produced per sample, of which over 28 million remained after quality assessment and filtering. 67% of the filtered reads aligned to the zebrafish reference genome GRCz11. Overall, 24436 genes were expressed in at least one of the three genotypes. Spearman’s rank correlation matrix of gene expression showed distinct clustering of 4 groups, resembling the 4 distinct genotypes: wild-type, *depdc5*, *tsc2* and double homozygotes (Figure 4A). Moreover, the analysis revealed a greater correlation between *tsc2* and *depdc5* larvae compared to double homozygous larvae.

Differential gene expression analysis showed a strong relation between the number of DEGs and the level of pS6, represented by an increase of pS6 levels compared to wild-types (Figure 4B). In fact, *depdc5* zebrafish larvae, having the lowest pS6 levels, displayed 704 DEGs compared to wild-types, of which 294 were down and 410 were upregulated (Figure 4C; Supplementary Table S2), whereas, *tsc2* zebrafish larvae, having higher pS6 levels, displayed 3177 DEGs compared to wild-types, of which 1370 were down and 1807 were upregulated genes (Figure 4D; Supplementary Table S2). Accordingly, double homozygous zebrafish larvae, expressing the highest pS6 levels of all genotypes, displayed 9349 DEGs compared to wild-types, of which 4644 were down and 4705 were upregulated (Figure 4E; Supplementary Table S2). Comparison of the DEGs of the different genotypes, demonstrated that a great percentage of DEGs of *depdc5* and *tsc2* homozygotes (respectively 90% and 80%) were differentially expressed in double homozygotes as well (Figure 4F).

To further interpret the biological implications of the large number of DEGs, pathway and GO enrichment analysis was performed. Generally, all three genotypes demonstrated that both aminoacyl tRNA synthesis and amino acid biosynthesis, which are biological processes directly related to mTORC1 hyperactivity, were enriched amongst differentially expressed genes (Supplementary Table S3). Subsequently, pathway and GO enrichment analysis of the transcriptome of the double homozygotes (Figures 4G,H) identified 19 enriched pathways amongst upregulated genes in double homozygotes versus wild-types, which were predominantly related to catabolism and mitochondrial functioning. For the downregulated genes, 10 enriched pathways were identified, amongst which the notch signalling pathway, a key player in neurodevelopmental processes. GO enrichment analysis showed a large number of GO terms enriched amongst both up and downregulated genes in the double homozygotes versus wild-type (Supplementary Table S3). Enrichment map analysis of the top 20 GO terms (Biological Process and Cellular Component) of upregulated genes demonstrated clusters related to ER stress, predominantly stimulating catabolism by the proteasome and inhibiting translation, and other nucleus-, Golgi apparatus- and mitochondrial-related processes (Figure 4H, right side). For the downregulated genes, most top 20 GO terms belonged to the two big clusters related to neuronal morphogenesis/projections and phototransduction (Figure 4H, left side).

Finally, enriched GO terms identified in *tsc2* and double homozygous zebrafish were compared (Figures 4H,I). Similarly to double homozygotes, Enrichment Map analysis of the top 10 enriched GO terms amongst upregulated genes in the *tsc2* homozygotes versus wild-types demonstrated clusters related to cellular stress levels (response to nitrogen compound), mitochondria and autophagy/lysosomes. Nevertheless, the mitochondrial-related cluster identified in the *tsc2* homozygotes was more restricted to the respiratory chain (RC) while in double homozygotes the cluster also encompasses terms related to mitochondrial organisation. Moreover, autophagy-related terms amongst upregulated genes were less significantly enriched in the double homozygotes compared to the *tsc2* homozygotes (Supplementary Tables S3, S4). Remarkably, the cluster related to synapses and synaptic transmission was enriched among upregulated genes for *tsc2* homozygotes, while it was enriched amongst downregulated genes for the double homozygotes. Additionally, a cell cycle-related cluster was only enriched in the *tsc2* homozygotes.

For RT-qPCR validation of the RNA-sequencing data, a set of 10 genes was used. Amongst upregulated genes, genes belonging to aminoacyl tRNA synthesis (*farsa1*)*,* inflammatory (*rnf14* and *ctsh*) and mitochondrial (*stoml2* and *phb*) pathways were selected. For downregulated genes, genes belonging to Notch signalling (*neurl1* and *hdac5*), synaptic transmission (*tspoap1*) and extracellular matrix (*reelin and col28a*) were selected. Altogether, the expression pattern of all selected genes mirrored the results obtained by RNA seq (Supplementary Figures S2A, B).

### Mitochondrial abnormalities in double homozygous zebrafish larvae overlap with human SEGA lesions

**FIGURE 5:**
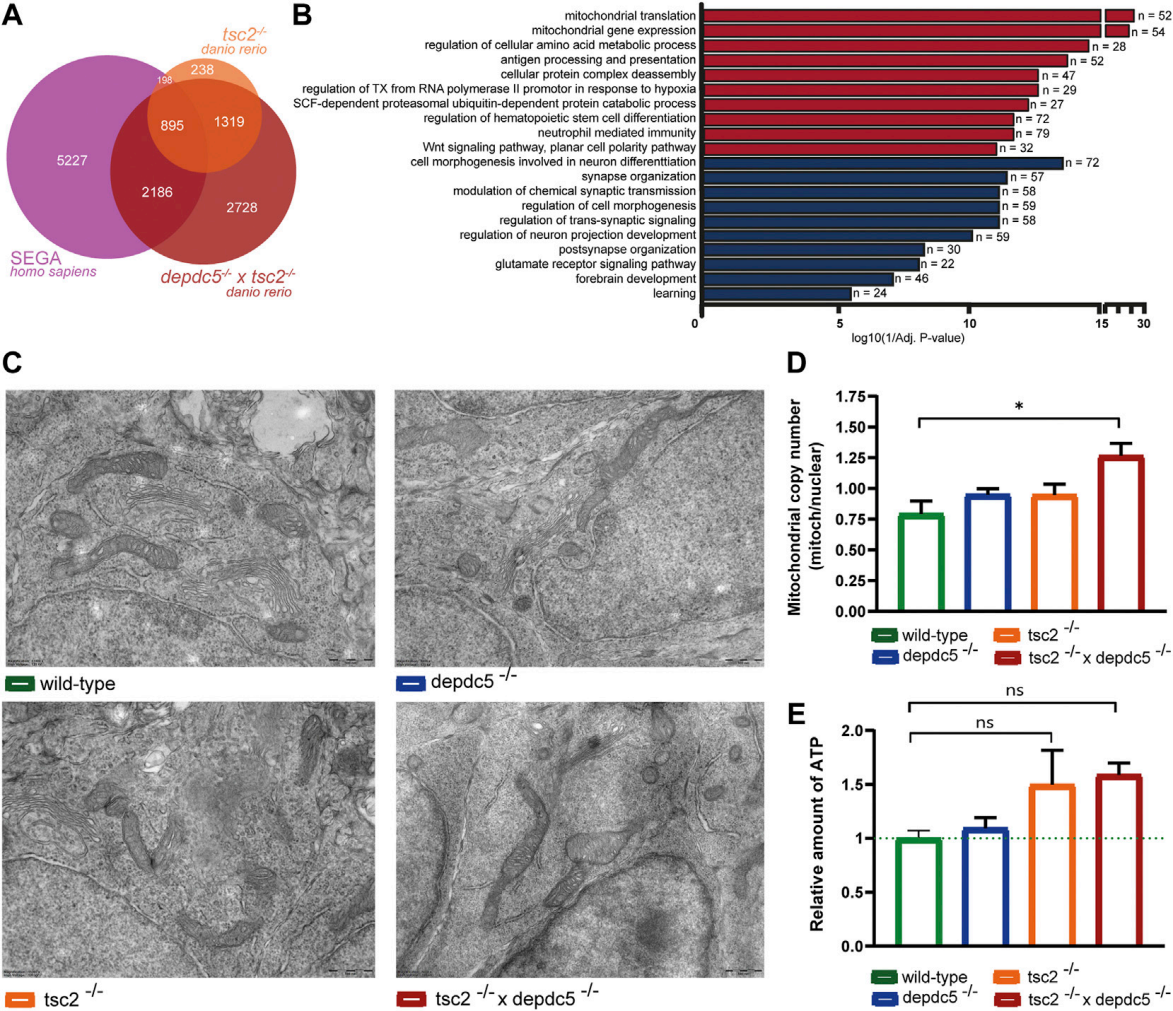
Comparison of the transcriptome of double homozygous zebrafish larvae and human SEGA samples. **(A)** Overlap of differentially expressed genes between *tsc2* ^*−/−*^ zebrafish larvae*,* double homozygous zebrafish larvae (after conversion to human orthologs) and SEGA samples. 38% and 13% of total DEGs in the SEGA transcriptome were found to overlap with the double and tsc2 homozygotes transcriptome, respectively. A Chi-square test indicated that there was a statistically significant association (*p* = 2.2e-16) between the zebrafish genotype and the proportion of overlapping genes. **(B)** Enriched GO terms from up and downregulated genes that are shared between double homozygous zebrafish larvae and human SEGA samples. Terms enriched amongst upregulated genes are indicated in red, terms enriched amongst downregulated genes are indicated in blue. The x-axis represents the log10 (1/*p*-value), n indicates the number of genes appearing in each category. **(C)** Representative electron microscopy images (scale bar; 500 nm) visualizing mitochondria in wild-type, *depdc5* ^−/−^, *tsc2* ^*−/−*^ *,* and double homozygous zebrafish larvae at 5 dpf. *n* = 1 larvae/group **(D)** Mitochondrial copy number in wild-type, *depdc5* ^−/−^, *tsc2* ^*−/−*^ *,* and double homozygous zebrafish heads at 5 dpf. Data are presented as mean ± SEM, *n* = 3–6 larvae/condition. Significant values (one-way ANOVA) are noted as *p < 0.05. **(E)** ATP Levels in wild-type, *depdc5* ^−/−^, *tsc2* ^*−/−*^ *,* and double homozygous zebrafish heads at 5 dpf. Data are presented as mean ± SEM, *n* = 15–20 larvae/condition. No statistical significant differences were detected (one-way ANOVA).

As second hit mutations are most frequently detected in SEN/SEGA lesions (Martin et al., 2017), the transcriptome of the double homozygous larvae and human SEGA lesions (Bongaarts et al., 2020) was compared to identify clinically relevant mechanisms. Both datasets displayed a similar amount of DEGs. Significantly, a substantial amount (38%) of the DEGs present in SEGA lesions was also found in the double homozygous zebrafish larvae, while in contrast, the transcriptome analysis of SEGA and *tsc2* homozygotes revealed a lower overlap, i.e., 13%. (Figure 5A, *p* < 2.2e-16). Next, pathway enrichment analysis of the overlapping genes revealed terms related to the immune system to be enriched amongst upregulated genes and terms related to synaptic (glutamatergic) signalling and cell morphology and neuronal projections enriched amongst downregulated genes. Additionally, pathways related to neurodevelopment, Notch and Wnt-signalling showed to be enriched amongst down and upregulated DEGs respectively. Interestingly, mitochondrial aspects (mitochondrial translation and mitochondrial gene expression) were again strongly enriched amongst the overlapping upregulated genes (Figure 5B; Supplementary Table S5).

To confirm whether mitochondrial genes were significantly overrepresented in the zebrafish and SEGA transcriptional profiles, several DEG lists (*tsc2* ^*−/−*^ *, depdc5* ^*−/−*^ *x tsc2* ^*−/−*^ *,* SEGA, overlap of *tsc2* ^*−/−*^ and SEGA, overlap of *depdc5* ^*−/−*^ *x tsc2* ^*−/−*^ and SEGA) were compared to the Human MitoCarta3.0 dataset (Rath et al., 2021), consisting of an extensive list of nuclear and mtDNA genes encoding proteins with strong support of mitochondrial localization. Hypergeometric testing showed a significant increment of mitochondrial genes within the SEGA transcriptome and in the overlap of DEGs in double homozygous zebrafish and SEGA (respectively *p* = 1.09518e-10 and *p* < 1.427117e-05, Supplementary Table S6).

Consequently, we examined ultrastructural aspects of the mitochondria by electron microscopy. In general, no gross mitochondrial abnormalities were observed at 5 dpf (Figure 5C). Next, the mitochondrial copy number was investigated by extraction of mitochondrial DNA and, as expected, only the double homozygotes displayed a significant increase in mitochondrial content compared to wild-type larvae (Figure 5D, *p* < 0.01). Subsequently, the mitochondrial function was assessed by measuring the ATP levels in all homozygotes. This functional analysis revealed a trend towards increased ATP levels in the *tsc2* and double homozygotes (Figure 5E, *p* > 0.05).

Finally, to illustrate the value of the double mutant zebrafish model in the identification of possible new pharmacological targets for SEGA, the overlapping DEGs of the double mutant zebrafish and human SEGA transcriptome were subjected to drug-gene interaction analysis using the DGIdb database. Of 2061 overlapping genes, 339 were associated with 4439 drug-gene interactions. We found numerous MAPK inhibitors, but also mTOR inhibitors amongst the reported interactions.

## Discussion

To assess the biological effects of hyperactive mTORC1 *in vivo* conditions, we were interested in a double mutant zebrafish model that carries mutations both in the *tsc2* and another relevant mTOR-related gene. Significantly, gain of function (GOF) mutations have been described in *RHEB, MTOR* and *RPS6* (Pelorosso et al., 2019; Zhao et al., 2019). However, corresponding GOF models in zebrafish have yet to be generated and their phenotype with anticipated stimulatory effects on the mTORC1 pathway to be fully characterized. On the other hand, loss of function (LOF) mutations have been discovered in *TSC1* and *DEPDC5* (Martin et al., 2017; Sim et al., 2019). For example, combined *TSC1* and *TSC2* mutations have been detected in patients and functionally characterized using a *TSC1* ^*fl/-*^ *;hGFAP-Cre x TSC2* ^*fl/-*^ *;hGFAP-Cre* mouse model. Unfortunately, these double mutant mice did not show an enhanced activation of mTORC1 compared to the single mutants (Mietzsch et al., 2013), an outcome that is likely explained by the strong interdependence of the proteins encoded by the TSC1 (hamartin) and TSC2 (tuberin) genes for the stability and functioning of the TSC complex. In addition, also the generation of a *tsc1* zebrafish mutant is complicated by the presence of two zebrafish paralogues for the human *TSC1* gene.

As a result, we opted for a double mutant zebrafish model that carries a LOF mutation both in the *tsc2* and the *depdc5* gene. To the best of our knowledge, this is the first time a second-hit model has been studied in zebrafish. Significantly, the *DEPDC5* gene has an important role in the regulation of mTORC1 (Shimobayashi and Hall, 2016), and the combination of a germline *TSC2* and somatic *DEPDC5* mutation has been described in a TSC patient with intractable epilepsy (Sim et al., 2019). Finally, a *depdc5* LOF zebrafish model has been extensively characterized for brain-related manifestations and hyperactivity of mTORC1 has been observed (Swaminathan et al., 2018).

As anticipated, by interfering with two distinct negative regulatory arms of the mTORC1 pathway, we found hyperactivation of mTORC1 in the double homozygotes that exceeded those found in the *depdc5* and *tsc2* mutants. The double homozygotes showed premature lethality that could be almost completely reversed by rapamycin treatment, an outcome that has also been observed in other TSC models (Meikle et al., 2007; Zeng et al., 2008; Di Nardo et al., 2009; Goto et al., 2011; Magri et al., 2011; Magri et al., 2013) and which unequivocally identifies the enhanced mTORC1 effects as the cause of the biological response. Evidently, because we used a global KO strategy that affected all cells, a more dramatic consequence was expected compared to a conditional KO strategy used in some *TSC*-deficient animal models where the loss of *TSC1/2* was limited to a specific number of cells from a specific point in time. However, this outcome also demonstrates the power of *ex utero* developing zebrafish as a model system to study pathogenic processes (Rosch et al., 2019) especially when compared to global KO mouse models where *in utero* development in combination with a possible early lethal phenotype hinders a full study of the disease.

In line with other mTOR-related models with shorter lifespan (Yu and Cui, 2016), the strong mTORC1 dysregulation in the double homozygotes translated into a clear reduction of their survival compared to the *tsc2* homozygotes. This result can be explained by a dramatic disruption of cellular homeostasis, as inappropriate nutrient sensing, anabolism and reduction of autophagy are all affected by mTORC1 hyperactivity with a documented negative effect on lifespan (Weichhart, 2018). In fact, the transcriptional profile of the double homozygotes reveals more anabolic metabolism supporting cell proliferation compared to the *tsc2* homozygotes. Accordingly, at transcriptional level GO terms related to translation are upregulated amongst the downregulated DEGs in the tsc2 homozygotes. In the double homozygous zebrafish larvae, the situation is more complex with GO terms as “peptide biosynthetic process” and “protein catabolic process” that are significantly and strongly upregulated amongst the upregulated DEGs. Thus, it is likely that in the double homozygotes these biological processes keep each other in balance, which explains the total protein levels found that are similar to those in the wild-types. The results underline the divergent phenotypes resulting from the different degrees of mTORC1 hyperactivity. Also, the mitochondrial copy number in the double homozygotes was upregulated, likely to cope with higher cellular ATP demands as reported in certain cancers (Reznik et al., 2016). In addition, as mitochondria are the major contributors to the production of reactive oxygen species (ROS), the increased mitochondrial functioning in our *tsc2* and double homozygotes might result in oxidative stress (OS). This is supported by our transcriptional findings from the tsc2 and double homozygote ZF that showed an upregulation of NFE2-like BZIP transcription factor 2 (*NFE2L2*) and heme oxygenase 1 (*HMOX1*) (the later only in double homozygotes), a downregulation of catalase (*CAT*), and enrichment of the GO cell redox homeostasis which are all indicative of OS. Moreover, also human epileptogenic cortical samples and *TSC1/2*-deficient models showed increased OS levels (Nie et al., 2015; Arena et al., 2019), suggesting that OS contributes to the disease pathology, a mechanism conserved in zebrafish. This, combined with a clear indication of compromised catabolism as shown by reduced autophagy-related GO terms in double homozygotes compared to *tsc2* homozygotes, infers a highly disturbed metabolism present in the double homozygotes.

When investigating seizure-related behavioral aspects and electrographic signatures, we observed that the *tsc2* and double homozygotes exhibited spontaneous locomotor hypoactivity when exposed to a dark-light cycle, but both became equally hyperactive after the treatment with 5 mM PTZ, a GABA-antagonist. Conversely, the *depdc5* homozygotes showed behavioral responses in line with control larvae. In case of the double homozygotes, these behavioral changes were clearly supported by abnormal brain discharges, as evidenced by increased PSD-values, while this was not the case for the *tsc2* homozygotes. It is likely that the *tsc2* homozygotes displayed fewer generalized electrographic seizures as compared to their double homozygous counterparts that consequently were not recorded by the electrode monitoring signals generated only by a small population of superficial optic tectal neurons. A similar inconsistency between the outcome of locomotor assays and brain recordings have been reported before (Zhang et al., 2017).

Despite reports of neuronal hyperactivity in *TSC*-deficient models (Uhlmann et al., 2002; Goto et al., 2011; Magri et al., 2011; Magri et al., 2013; Winden et al., 2019) spontaneous epileptiform activity was not detected in the *tsc2* nor in the double homozygous larvae. While somewhat surprising, our results are in line with a recent study showing that of the 40 single-gene mutant zebrafish lines representing catastrophic childhood epilepsies, only 20% exhibited unprovoked electrographic seizures (Griffin et al., 2021). In contrast, as revealed by the results obtained with subthreshold concentrations of PTZ, the double homozygotes showed augmented seizure susceptibility. As epilepsy is considered a disease of abnormal brain networks, our findings can be explained by the role of mTORC1 in the formation of neuronal circuits. Of interest, between 2-3 dpf zebrafish experience a second wave of neurogenesis in which the basic neuronal network is replaced by a mature and more extensive neuronal circuitry (Tropepe and Sive, 2003). Since we showed that the mTORC1 activity became highly active during the 3-5 dpf period, and increased mTORC1 activity results in accelerated axon growth (Gong et al., 2015) and guidance defects (Nie et al., 2010), dendritic hypertrophy (Kumar et al., 2005; Tavazoie et al., 2005) and spine abnormalities (Kumar et al., 2005), it is tempting to speculate that mTORC1 hyperactivity resulted in pathological neuronal wiring diagrams in 5 dpf zebrafish brain and thus increased susceptibility to seizures. Accordingly, the transcriptional profile of double mutant demonstrated abnormalities in neuronal projection morphogenesis, regulation of neuronal projections and axon guidance. Moreover, mTORC1 is also thought to have indirect effects on the expression of ion channels and receptors, affecting the membrane potential (Raab-Graham et al., 2006; Li et al., 2010). In fact, GO enrichment analysis showed a reduction of GABAergic signalling in the double homozygous zebrafish compared to *tsc2* homozygotes which further might explain their hypersensitivity towards PTZ.

Our data on neuronal cell size and brain anatomy did not reveal differences in *depdc5* homozygotes compared to wild-type larvae, likely inferring that cellular and histopathological changes can only be expected above a threshold of mTORC1 activity. Accordingly, in the *tsc2* and double homozygotes enlarged cell size and ventricular dilatation were noticed. Dilatation of the brain ventricles is a feature occurring in humans with SEGA lesions (Mei et al., 2017) and rodent TSC models displaying SEGA-alike lesions (Magri et al., 2011; Zhou et al., 2011; Magri et al., 2013). Surprisingly, despite the varying degrees of mTORC1 hyperactivity between these mutants, no major histological differences were detected. We hypothesize that the limited time available (i.e., 5 dpf) before the double homozygotes became moribund, dramatically impeded the development of more severe histopathological features such as giant cells and SEGA lesions. Accordingly, in other *TSC*-deficient models, giant cells and SEGA lesions were observed only after significant development time (Goto et al., 2011; Zordan et al., 2018; Nguyen et al., 2019).

A solid argument for the clinical relevance of the double homozygous zebrafish model is the strong overlap between the transcriptome of SEGA lesions with our double mutant model but less so with the *tsc2* homozygotes. In line with other studies, inflammatory and immune system-related GO terms were highly enriched among the upregulated genes (Martin et al., 2017; Bongaarts et al., 2020), while neurodevelopmental genes were downregulated (Bongaarts et al., 2017; Shin et al., 2017). Interestingly, the transcriptional profile of the double homozygotes, as well as the overlap with SEGA lesions, demonstrated signs of dysregulated Notch signaling. Appropriate Notch signalling is pivotal for brain development as it influences neuronal differentiation, among other crucial processes (Lasky and Wu, 2005). Significantly, tubers and SEGA lesions encompass cells with dysregulated neuroglial expression patterns, and reduced expression of the Notch1 receptor has been observed in an astrocyte-specific *TSC1* conditional KO mice model (Ess et al., 2004). Moreover, analysis with DGIdb, an R package that integrates available data sources describing drug-gene interactions and druggable genes, identified numerous interactions with inhibitors of MAPK signaling (e.g., trametinib, MEK1/2 inhibitor). This outcome together with reports of activated MAPK signaling in SEGA lesions (Jozwiak et al., 2007; Tyburczy et al., 2010; Siedlecka et al., 2015; Bongaarts et al., 2020) make inhibition of these pathways a possible new and compelling clinical treatment option for double hit lesions. Significantly, mTOR inhibitors, the only approved pharmacological therapy for SEGA, were also observed in the gene-drug list, further validating our approach and supporting the potential of our model to detect clinically relevant therapies.

In conclusion, we demonstrate a strong relationship between the degree of mTORC1 hyperactivity and number of DEGs and thus a proportionally stronger disruption of normal development in the double homozygous larvae. Accordingly, the double homozygotes displayed a higher seizure susceptibility which might indicate a role for second hit mutations in the exacerbation of the clinical epilepsy phenotype. Conversely, no gross histological differences were observed, while the comparison of the transcriptomes of the double homozygous larvae and human SEGA lesions revealed strong overlap. As strong pS6 labeling has been reported in SEGA lesions, we found further evidence for a causal role of hyperactive mTORC1 in SEGA formation. Moreover, by analysis of gene-drug interactions we identified inhibitors of the MAPK pathways as possible interesting pharmacological tools for the treatment of SEGA lesions. Taken together, the data show that our double mutated zebrafish model could be cost-effective in value for future drug discovery efforts.

## Data Availability

The datasets presented in this study can be found in online repositories. The names of the repository/repositories and accession number(s) can be found below: The RNA sequencing data have been uploaded to the ArrayExpress repository (with the accession number: E-MTAB-11776).

## References

[B1] Afrikanova T. Serruys A. S. Buenafe O. E. Clinckers R. Smolders I. De Witte P. A. (2013). Validation of the zebrafish pentylenetetrazol seizure model: Locomotor versus electrographic responses to antiepileptic drugs. PLoS One 8, e54166. 10.1371/journal.pone.0054166 23342097 PMC3544809

[B2] Anderson M. P. (2018). DEPDC5 takes a second hit in familial focal epilepsy. J. Clin. Invest. 128, 2194–2196. 10.1172/JCI121052 29708509 PMC5983308

[B3] Arena A. Zimmer T. S. Van Scheppingen J. Korotkov A. Anink J. J. Muhlebner A. (2019). Oxidative stress and inflammation in a spectrum of epileptogenic cortical malformations: Molecular insights into their interdependence. Brain Pathol. 29, 351–365. 10.1111/bpa.12661 30303592 PMC8028690

[B4] Artuso L. Romano A. Verri T. Domenichini A. Argenton F. Santorelli F. M. (2012). Mitochondrial DNA metabolism in early development of zebrafish (*Danio rerio*). Biochim. Biophys. Acta 1817, 1002–1011. 10.1016/j.bbabio.2012.03.019 22465854

[B5] Ashburner M. Ball C. A. Blake J. A. Botstein D. Butler H. Cherry J. M. (2000). Gene ontology: Tool for the unification of biology. The gene ontology consortium. Nat. Genet. 25, 25–29. 10.1038/75556 10802651 PMC3037419

[B6] Baldassari S. Ribierre T. Marsan E. Adle-Biassette H. Ferrand-Sorbets S. Bulteau C. (2019). Dissecting the genetic basis of focal cortical dysplasia: A large cohort study. Acta Neuropathol. 138, 885–900. 10.1007/s00401-019-02061-5 31444548 PMC6851393

[B7] Ben-Sahra I. Manning B. D. (2017). mTORC1 signaling and the metabolic control of cell growth. Curr. Opin. Cell Biol. 45, 72–82. 10.1016/j.ceb.2017.02.012 28411448 PMC5545101

[B8] Bolger A. M. Lohse M. Usadel B. (2014). Trimmomatic: A flexible trimmer for illumina sequence data. Bioinformatics 30, 2114–2120. 10.1093/bioinformatics/btu170 24695404 PMC4103590

[B9] Bongaarts A. Giannikou K. Reinten R. J. Anink J. J. Mills J. D. Jansen F. E. (2017). Subependymal giant cell astrocytomas in Tuberous Sclerosis Complex have consistent TSC1/TSC2 biallelic inactivation, and no BRAF mutations. Oncotarget 8, 95516–95529. 10.18632/oncotarget.20764 29221145 PMC5707039

[B10] Bongaarts A. Van Scheppingen J. Korotkov A. Mijnsbergen C. Anink J. J. Jansen F. E. (2020). The coding and non-coding transcriptional landscape of subependymal giant cell astrocytomas. Brain 143, 131–149. 10.1093/brain/awz370 31834371 PMC6935755

[B11] Byrnes J. Ganetzky R. Lightfoot R. Tzeng M. Nakamaru-Ogiso E. Seiler C. (2018). Pharmacologic modeling of primary mitochondrial respiratory chain dysfunction in zebrafish. Neurochem. Int. 117, 23–34. 10.1016/j.neuint.2017.07.008 28732770 PMC5773416

[B12] Chen E. Y. Tan C. M. Kou Y. Duan Q. Wang Z. Meirelles G. V. (2013). Enrichr: Interactive and collaborative HTML5 gene list enrichment analysis tool. BMC Bioinforma. 14, 128. 10.1186/1471-2105-14-128 PMC3637064 23586463

[B13] Costa V. Aigner S. Vukcevic M. Sauter E. Behr K. Ebeling M. (2016). mTORC1 inhibition corrects neurodevelopmental and synaptic alterations in a human stem cell model of tuberous sclerosis. Cell Rep. 15, 86–95. 10.1016/j.celrep.2016.02.090 27052171

[B14] Crino P. B. Aronica E. Baltuch G. Nathanson K. L. (2010). Biallelic TSC gene inactivation in tuberous sclerosis complex. Neurology 74, 1716–1723. 10.1212/WNL.0b013e3181e04325 20498439 PMC2882213

[B15] Curatolo P. Moavero R. De Vries P. J. (2015a). Neurological and neuropsychiatric aspects of tuberous sclerosis complex. Lancet. Neurol. 14, 733–745. 10.1016/S1474-4422(15)00069-1 26067126

[B16] Curatolo P. Moavero R. Roberto D. Graziola F. (2015b). Genotype/phenotype correlations in tuberous sclerosis complex. Semin. Pediatr. Neurol. 22, 259–273. 10.1016/j.spen.2015.10.002 26706013

[B17] Curatolo P. Moavero R. Van Scheppingen J. Aronica E. (2018). mTOR dysregulation and tuberous sclerosis-related epilepsy. Expert Rev. Neurother. 18, 185–201. 10.1080/14737175.2018.1428562 29338461

[B18] D'gama A. M. Woodworth M. B. Hossain A. A. Bizzotto S. Hatem N. E. Lacoursiere C. M. (2017). Somatic mutations activating the mTOR pathway in dorsal telencephalic progenitors cause a continuum of cortical dysplasias. Cell Rep. 21, 3754–3766. 10.1016/j.celrep.2017.11.106 29281825 PMC5752134

[B19] Di Nardo A. Kramvis I. Cho N. Sadowski A. Meikle L. Kwiatkowski D. J. (2009). Tuberous sclerosis complex activity is required to control neuronal stress responses in an mTOR-dependent manner. J. Neurosci. 29, 5926–5937. 10.1523/JNEUROSCI.0778-09.2009 19420259 PMC2691854

[B20] Dobin A. Davis C. A. Schlesinger F. Drenkow J. Zaleski C. Jha S. (2013). Star: Ultrafast universal RNA-seq aligner. Bioinformatics 29, 15–21. 10.1093/bioinformatics/bts635 23104886 PMC3530905

[B21] Ess K. C. Uhlmann E. J. Li W. Li H. Declue J. E. Crino P. B. (2004). Expression profiling in tuberous sclerosis complex (TSC) knockout mouse astrocytes to characterize human TSC brain pathology. Glia 46, 28–40. 10.1002/glia.10324 14999811

[B22] Freshour S. L. Kiwala S. Cotto K. C. Coffman A. C. Mcmichael J. F. Song J. J. (2021). Integration of the drug-gene interaction database (DGIdb 4.0) with open crowdsource efforts. Nucleic Acids Res. 49, D1144–D1151. 10.1093/nar/gkaa1084 33237278 PMC7778926

[B23] Gong X. Zhang L. Huang T. Lin T. V. Miyares L. Wen J. (2015). Activating the translational repressor 4E-BP or reducing S6K-GSK3β activity prevents accelerated axon growth induced by hyperactive mTOR *in vivo*. Hum. Mol. Genet. 24, 5746–5758. 10.1093/hmg/ddv295 26220974 PMC4581604

[B24] Goto J. Talos D. M. Klein P. Qin W. Chekaluk Y. I. Anderl S. (2011). Regulable neural progenitor-specific Tsc1 loss yields giant cells with organellar dysfunction in a model of tuberous sclerosis complex. Proc. Natl. Acad. Sci. U. S. A. 108, E1070–E1079. 10.1073/pnas.1106454108 22025691 PMC3214999

[B25] Griffin A. Carpenter C. Liu J. Paterno R. Grone B. Hamling K. (2021). Phenotypic analysis of catastrophic childhood epilepsy genes. Commun. Biol. 4, 680. 10.1038/s42003-021-02221-y 34083748 PMC8175701

[B26] Henske E. P. Jozwiak S. Kingswood J. C. Sampson J. R. Thiele E. A. (2016). Tuberous sclerosis complex. Nat. Rev. Dis. Prim. 2, 16035. 10.1038/nrdp.2016.35 27226234

[B27] Hornberg H. Cioni J. M. Harris W. A. Holt C. E. (2016). Hermes regulates axon sorting in the optic tract by post-trancriptional regulation of neuropilin 1. J. Neurosci. 36, 12697–12706. 10.1523/JNEUROSCI.2400-16.2016 27974617 PMC5157111

[B28] Hulshof H. M. Kuijf H. J. Kotulska K. Curatolo P. Weschke B. Riney K. (2022). Association of early MRI characteristics with subsequent epilepsy and neurodevelopmental outcomes in children with tuberous sclerosis complex. Neurology 98, e1216–e1225. 10.1212/WNL.0000000000200027 35101906

[B29] Hunter S. E. Jung D. Di Giulio R. T. Meyer J. N. (2010). The QPCR assay for analysis of mitochondrial DNA damage, repair, and relative copy number. Methods 51, 444–451. 10.1016/j.ymeth.2010.01.033 20123023 PMC2912960

[B30] Isserlin R. Merico D. Voisin V. Bader G. D. (2014). Enrichment Map - a Cytoscape app to visualize and explore OMICs pathway enrichment results. F1000Res. 3, 141. 10.12688/f1000research.4536.1 25075306 PMC4103489

[B31] Jozwiak J. Grajkowska W. Kotulska K. Jozwiak S. Zalewski W. Zajaczkowska A. (2007). Brain tumor formation in tuberous sclerosis depends on Erk activation. Neuromolecular Med. 9, 117–127. 10.1007/BF02685886 17627032

[B32] Kanehisa M. Goto S. (2000). Kegg: Kyoto encyclopedia of genes and genomes. Nucleic Acids Res. 28, 27–30. 10.1093/nar/28.1.27 10592173 PMC102409

[B33] Kedra M. Banasiak K. Kisielewska K. Wolinska-Niziol L. Jaworski J. Zmorzynska J. (2020). TrkB hyperactivity contributes to brain dysconnectivity, epileptogenesis, and anxiety in zebrafish model of Tuberous Sclerosis Complex. Proc. Natl. Acad. Sci. U. S. A. 117, 2170–2179. 10.1073/pnas.1910834117 31932427 PMC6995026

[B34] Kim S. H. Speirs C. K. Solnica-Krezel L. Ess K. C. (2011). Zebrafish model of tuberous sclerosis complex reveals cell-autonomous and non-cell-autonomous functions of mutant tuberin. Dis. Model. Mech. 4, 255–267. 10.1242/dmm.005587 20959633 PMC3046101

[B35] Kumar V. Zhang M. X. Swank M. W. Kunz J. Wu G. Y. (2005). Regulation of dendritic morphogenesis by Ras-PI3K-Akt-mTOR and Ras-MAPK signaling pathways. J. Neurosci. 25, 11288–11299. 10.1523/JNEUROSCI.2284-05.2005 16339024 PMC6725910

[B36] Lasky J. L. Wu H. (2005). Notch signaling, brain development, and human disease. Pediatr. Res. 57, 104R–109R. 10.1203/01.PDR.0000159632.70510.3D 15817497

[B37] Li N. Lee B. Liu R. J. Banasr M. Dwyer J. M. Iwata M. (2010). mTOR-dependent synapse formation underlies the rapid antidepressant effects of NMDA antagonists. Science 329, 959–964. 10.1126/science.1190287 20724638 PMC3116441

[B38] Li J. Copmans D. Partoens M. Hunyadi B. Luyten W. De Witte P. (2020). Zebrafish-based screening of antiseizure plants used in traditional Chinese medicine: Magnolia officinalis extract and its constituents magnolol and honokiol exhibit potent anticonvulsant activity in a therapy-resistant epilepsy model. ACS Chem. Neurosci. 11, 730–742. 10.1021/acschemneuro.9b00610 32083464

[B39] Liao Y. Smyth G. K. Shi W. (2014). featureCounts: an efficient general purpose program for assigning sequence reads to genomic features. Bioinformatics 30, 923–930. 10.1093/bioinformatics/btt656 24227677

[B40] Louis D. N. Perry A. Wesseling P. Brat D. J. Cree I. A. Figarella-Branger D. (2021). The 2021 WHO classification of tumors of the central nervous system: A summary. Neuro. Oncol. 23, 1231–1251. 10.1093/neuonc/noab106 34185076 PMC8328013

[B41] Love M. I. Huber W. Anders S. (2014). Moderated estimation of fold change and dispersion for RNA-seq data with DESeq2. Genome Biol. 15, 550. 10.1186/s13059-014-0550-8 25516281 PMC4302049

[B42] Lu D. S. Karas P. J. Krueger D. A. Weiner H. L. (2018). Central nervous system manifestations of tuberous sclerosis complex. Am. J. Med. Genet. C Semin. Med. Genet. 178, 291–298. 10.1002/ajmg.c.31647 30230171

[B43] Macrae C. A. Peterson R. T. (2015). Zebrafish as tools for drug discovery. Nat. Rev. Drug Discov. 14, 721–731. 10.1038/nrd4627 26361349

[B44] Magri L. Cambiaghi M. Cominelli M. Alfaro-Cervello C. Cursi M. Pala M. (2011). Sustained activation of mTOR pathway in embryonic neural stem cells leads to development of tuberous sclerosis complex-associated lesions. Cell Stem Cell 9, 447–462. 10.1016/j.stem.2011.09.008 22056141

[B45] Magri L. Cominelli M. Cambiaghi M. Cursi M. Leocani L. Minicucci F. (2013). Timing of mTOR activation affects tuberous sclerosis complex neuropathology in mouse models. Dis. Model. Mech. 6, 1185–1197. 10.1242/dmm.012096 23744272 PMC3759338

[B46] Martin K. R. Zhou W. Bowman M. J. Shih J. Au K. S. Dittenhafer-Reed K. E. (2017). The genomic landscape of tuberous sclerosis complex. Nat. Commun. 8, 15816. 10.1038/ncomms15816 28643795 PMC5481739

[B47] Mei G-H. Liu X-X. Zhou P. Shen M. (2017). Clinical and imaging features of subependymal giant cell astrocytoma: Report of 20 cases. Chin. Neurosurg. J. 3, 14. 10.1186/s41016-017-0077-4

[B48] Meikle L. Talos D. M. Onda H. Pollizzi K. Rotenberg A. Sahin M. (2007). A mouse model of tuberous sclerosis: Neuronal loss of Tsc1 causes dysplastic and ectopic neurons, reduced myelination, seizure activity, and limited survival. J. Neurosci. 27, 5546–5558. 10.1523/JNEUROSCI.5540-06.2007 17522300 PMC6672762

[B49] MietzschU.MckennaJ.3rdReithR. M.WayS. W.GambelloM. J. (2013). Comparative analysis of Tsc1 and Tsc2 single and double radial glial cell mutants. J. Comp. Neurol. 521, 3817–3831. 10.1002/cne.23380 23749404

[B50] Moavero R. Muhlebner A. Luinenburg M. J. Craiu D. Aronica E. Curatolo P. (2021). Genetic pathogenesis of the epileptogenic lesions in Tuberous Sclerosis Complex: Therapeutic targeting of the mTOR pathway. Epilepsy Behav. 131, 107713. 10.1016/j.yebeh.2020.107713 33431351

[B51] Muhlebner A. Bongaarts A. Sarnat H. B. Scholl T. Aronica E. (2019). New insights into a spectrum of developmental malformations related to mTOR dysregulations: Challenges and perspectives. J. Anat. 235, 521–542. 10.1111/joa.12956 30901081 PMC6704243

[B52] Nguyen L. H. Mahadeo T. Bordey A. (2019). mTOR hyperactivity levels influence the severity of epilepsy and associated neuropathology in an experimental model of tuberous sclerosis complex and focal cortical dysplasia. J. Neurosci. 39, 2762–2773. 10.1523/JNEUROSCI.2260-18.2019 30700531 PMC6445990

[B53] Nie D. Di Nardo A. Han J. M. Baharanyi H. Kramvis I. Huynh T. (2010). Tsc2-Rheb signaling regulates EphA-mediated axon guidance. Nat. Neurosci. 13, 163–172. 10.1038/nn.2477 20062052 PMC2812631

[B54] Nie D. Chen Z. Ebrahimi-Fakhari D. Di Nardo A. Julich K. Robson V. K. (2015). The stress-induced atf3-gelsolin cascade underlies dendritic spine deficits in neuronal models of tuberous sclerosis complex. J. Neurosci. 35, 10762–10772. 10.1523/JNEUROSCI.4796-14.2015 26224859 PMC4518051

[B55] Noble S. Godoy R. Affaticati P. Ekker M. (2015). Transgenic zebrafish expressing mCherry in the mitochondria of dopaminergic neurons. Zebrafish 12, 349–356. 10.1089/zeb.2015.1085 26355474

[B56] Pelorosso C. Watrin F. Conti V. Buhler E. Gelot A. Yang X. (2019). Somatic double-hit in MTOR and RPS6 in hemimegalencephaly with intractable epilepsy. Hum. Mol. Genet. 28, 3755–3765. 10.1093/hmg/ddz194 31411685 PMC6935386

[B57] Qin J. Wang Z. Hoogeveen-Westerveld M. Shen G. Gong W. Nellist M. (2016). Structural basis of the interaction between tuberous sclerosis complex 1 (TSC1) and tre2-bub2-cdc16 domain family member 7 (TBC1D7). J. Biol. Chem. 291, 8591–8601. 10.1074/jbc.M115.701870 26893383 PMC4861430

[B58] Raab-Graham K. F. Haddick P. C. Jan Y. N. Jan L. Y. (2006). Activity- and mTOR-dependent suppression of Kv1.1 channel mRNA translation in dendrites. Science 314, 144–148. 10.1126/science.1131693 17023663

[B59] Rath S. Sharma R. Gupta R. Ast T. Chan C. Durham T. J. (2021). MitoCarta3.0: An updated mitochondrial proteome now with sub-organelle localization and pathway annotations. Nucleic Acids Res. 49, D1541–D1547. 10.1093/nar/gkaa1011 33174596 PMC7778944

[B60] Reznik E. Miller M. L. Senbabaoglu Y. Riaz N. Sarungbam J. Tickoo S. K. (2016). Mitochondrial DNA copy number variation across human cancers. Elife 5, e10769. 10.7554/eLife.10769 26901439 PMC4775221

[B61] Ribierre T. Deleuze C. Bacq A. Baldassari S. Marsan E. Chipaux M. (2018). Second-hit mosaic mutation in mTORC1 repressor DEPDC5 causes focal cortical dysplasia-associated epilepsy. J. Clin. Invest. 128, 2452–2458. 10.1172/JCI99384 29708508 PMC5983335

[B62] Rosch R. Burrows D. R. W. Jones L. B. Peters C. H. Ruben P. Samarut E. (2019). Functional genomics of epilepsy and associated neurodevelopmental disorders using simple animal models: From genes, molecules to brain networks. Front. Cell. Neurosci. 13, 556. 10.3389/fncel.2019.00556 31920556 PMC6923670

[B63] Satou C. Kimura Y. Higashijima S. (2012). Generation of multiple classes of V0 neurons in zebrafish spinal cord: Progenitor heterogeneity and temporal control of neuronal diversity. J. Neurosci. 32, 1771–1783. 10.1523/JNEUROSCI.5500-11.2012 22302816 PMC6703360

[B64] Scheffer I. E. Heron S. E. Regan B. M. Mandelstam S. Crompton D. E. Hodgson B. L. (2014). Mutations in mammalian target of rapamycin regulator DEPDC5 cause focal epilepsy with brain malformations. Ann. Neurol. 75, 782–787. 10.1002/ana.24126 24585383

[B65] Scheldeman C. Mills J. D. Siekierska A. Serra I. Copmans D. Iyer A. M. (2017). mTOR-related neuropathology in mutant tsc2 zebrafish: Phenotypic, transcriptomic and pharmacological analysis. Neurobiol. Dis. 108, 225–237. 10.1016/j.nbd.2017.09.004 28888969

[B66] Shimobayashi M. Hall M. N. (2016). Multiple amino acid sensing inputs to mTORC1. Cell Res. 26, 7–20. 10.1038/cr.2015.146 26658722 PMC4816134

[B67] Shin J. Kim M. Jung H. J. Cha H. L. Suh-Kim H. Ahn S. (2017). Characterization of developmental defects in the forebrain resulting from hyperactivated mTOR signaling by integrative analysis of transcriptomic and proteomic data. Sci. Rep. 7, 2826. 10.1038/s41598-017-02842-6 28588230 PMC5460284

[B68] Siedlecka M. Szlufik S. Grajkowska W. Roszkowski M. Jozwiak J. (2015). Erk activation as a possible mechanism of transformation of subependymal nodule into subependymal giant cell astrocytoma. Folia Neuropathol. 53, 8–14. 10.5114/fn.2015.49969 25909870

[B69] Sim N. S. Ko A. Kim W. K. Kim S. H. Kim J. S. Shim K. W. (2019). Precise detection of low-level somatic mutation in resected epilepsy brain tissue. Acta Neuropathol. 138, 901–912. 10.1007/s00401-019-02052-6 31377847

[B70] Smedley D. Haider S. Durinck S. Pandini L. Provero P. Allen J. (2015). The BioMart community portal: An innovative alternative to large, centralized data repositories. Nucleic Acids Res. 43, W589–W598. 10.1093/nar/gkv350 25897122 PMC4489294

[B71] Stewart A. M. Gerlai R. Kalueff A. V. (2015). Developing highER-throughput zebrafish screens for *in-vivo* CNS drug discovery. Front. Behav. Neurosci. 9, 14. 10.3389/fnbeh.2015.00014 25729356 PMC4325915

[B72] Sundberg M. Tochitsky I. Buchholz D. E. Winden K. Kujala V. Kapur K. (2018). Purkinje cells derived from TSC patients display hypoexcitability and synaptic deficits associated with reduced FMRP levels and reversed by rapamycin. Mol. Psychiatry 23, 2167–2183. 10.1038/s41380-018-0018-4 29449635 PMC6093816

[B73] Swaminathan A. Hassan-Abdi R. Renault S. Siekierska A. Riche R. Liao M. (2018). Non-canonical mTOR-independent role of DEPDC5 in regulating GABAergic network development. Curr. Biol. 28, 1924–1937. e1925. 10.1016/j.cub.2018.04.061 29861134

[B74] Switon K. Kotulska K. Janusz-Kaminska A. Zmorzynska J. Jaworski J. (2017). Molecular neurobiology of mTOR. Neuroscience 341, 112–153. 10.1016/j.neuroscience.2016.11.017 27889578

[B75] Tavazoie S. F. Alvarez V. A. Ridenour D. A. Kwiatkowski D. J. Sabatini B. L. (2005). Regulation of neuronal morphology and function by the tumor suppressors Tsc1 and Tsc2. Nat. Neurosci. 8, 1727–1734. 10.1038/nn1566 16286931

[B76] Tropepe V. Sive H. L. (2003). Can zebrafish be used as a model to study the neurodevelopmental causes of autism? Genes Brain Behav. 2, 268–281. 10.1034/j.1601-183x.2003.00038.x 14606692

[B77] Tyburczy M. E. Kotulska K. Pokarowski P. Mieczkowski J. Kucharska J. Grajkowska W. (2010). Novel proteins regulated by mTOR in subependymal giant cell astrocytomas of patients with tuberous sclerosis complex and new therapeutic implications. Am. J. Pathol. 176, 1878–1890. 10.2353/ajpath.2010.090950 20133820 PMC2843477

[B78] Uhlmann E. J. Apicelli A. J. Baldwin R. L. Burke S. P. Bajenaru M. L. Onda H. (2002). Heterozygosity for the tuberous sclerosis complex (TSC) gene products results in increased astrocyte numbers and decreased p27-Kip1 expression in TSC2+/- cells. Oncogene 21, 4050–4059. 10.1038/sj.onc.1205435 12037687

[B79] Weichhart T. (2018). mTOR as regulator of lifespan, aging, and cellular senescence: A mini-review. Gerontology 64, 127–134. 10.1159/000484629 29190625 PMC6089343

[B80] Winden K. D. Sundberg M. Yang C. Wafa S. M. A. Dwyer S. Chen P. F. (2019). Biallelic mutations in TSC2 lead to abnormalities associated with cortical tubers in human iPSC-derived neurons. J. Neurosci. 39, 9294–9305. 10.1523/JNEUROSCI.0642-19.2019 31591157 PMC6867816

[B81] Yu G. He Q. Y. (2016). ReactomePA: An R/bioconductor package for reactome pathway analysis and visualization. Mol. Biosyst. 12, 477–479. 10.1039/c5mb00663e 26661513

[B82] Yu J. S. Cui W. (2016). Proliferation, survival and metabolism: The role of PI3K/AKT/mTOR signalling in pluripotency and cell fate determination. Development 143, 3050–3060. 10.1242/dev.137075 27578176

[B83] Zeng L. H. Xu L. Gutmann D. H. Wong M. (2008). Rapamycin prevents epilepsy in a mouse model of tuberous sclerosis complex. Ann. Neurol. 63, 444–453. 10.1002/ana.21331 18389497 PMC3937593

[B84] Zhang Y. Vanmeert M. Siekierska A. Ny A. John J. Callewaert G. (2017). Inhibition of glutamate decarboxylase (GAD) by ethyl ketopentenoate (EKP) induces treatment-resistant epileptic seizures in zebrafish. Sci. Rep. 7, 7195. 10.1038/s41598-017-06294-w 28775328 PMC5543107

[B85] Zhao S. Li Z. Zhang M. Zhang L. Zheng H. Ning J. (2019). A brain somatic RHEB doublet mutation causes focal cortical dysplasia type II. Exp. Mol. Med. 51, 84–11. 10.1038/s12276-019-0277-4 PMC6802736 31337748

[B86] Zhou J. Shrikhande G. Xu J. Mckay R. M. Burns D. K. Johnson J. E. (2011). Tsc1 mutant neural stem/progenitor cells exhibit migration deficits and give rise to subependymal lesions in the lateral ventricle. Genes Dev. 25, 1595–1600. 10.1101/gad.16750211 21828270 PMC3182017

[B87] Zordan P. Cominelli M. Cascino F. Tratta E. Poliani P. L. Galli R. (2018). Tuberous sclerosis complex-associated CNS abnormalities depend on hyperactivation of mTORC1 and Akt. J. Clin. Invest. 128, 1688–1706. 10.1172/JCI96342 29389670 PMC5873854

[B88] Zucco A. J. Pozzo V. D. Afinogenova A. Hart R. P. Devinsky O. D'arcangelo G. (2018). Neural progenitors derived from Tuberous Sclerosis Complex patients exhibit attenuated PI3K/AKT signaling and delayed neuronal differentiation. Mol. Cell. Neurosci. 92, 149–163. 10.1016/j.mcn.2018.08.004 30144504 PMC6250058

