# Supplementary figures and images for: Hyperactivation of mTORC1 in a double hit mutant zebrafish model of tuberous sclerosis complex causes increased seizure susceptibility and neurodevelopmental abnormalities
Source: Front Cell Dev Biol. 2022 Sep 27;10:952832. doi: 10.3389/fcell.2022.952832 (PMC9552079; doi:10.3389/fcell.2022.952832)

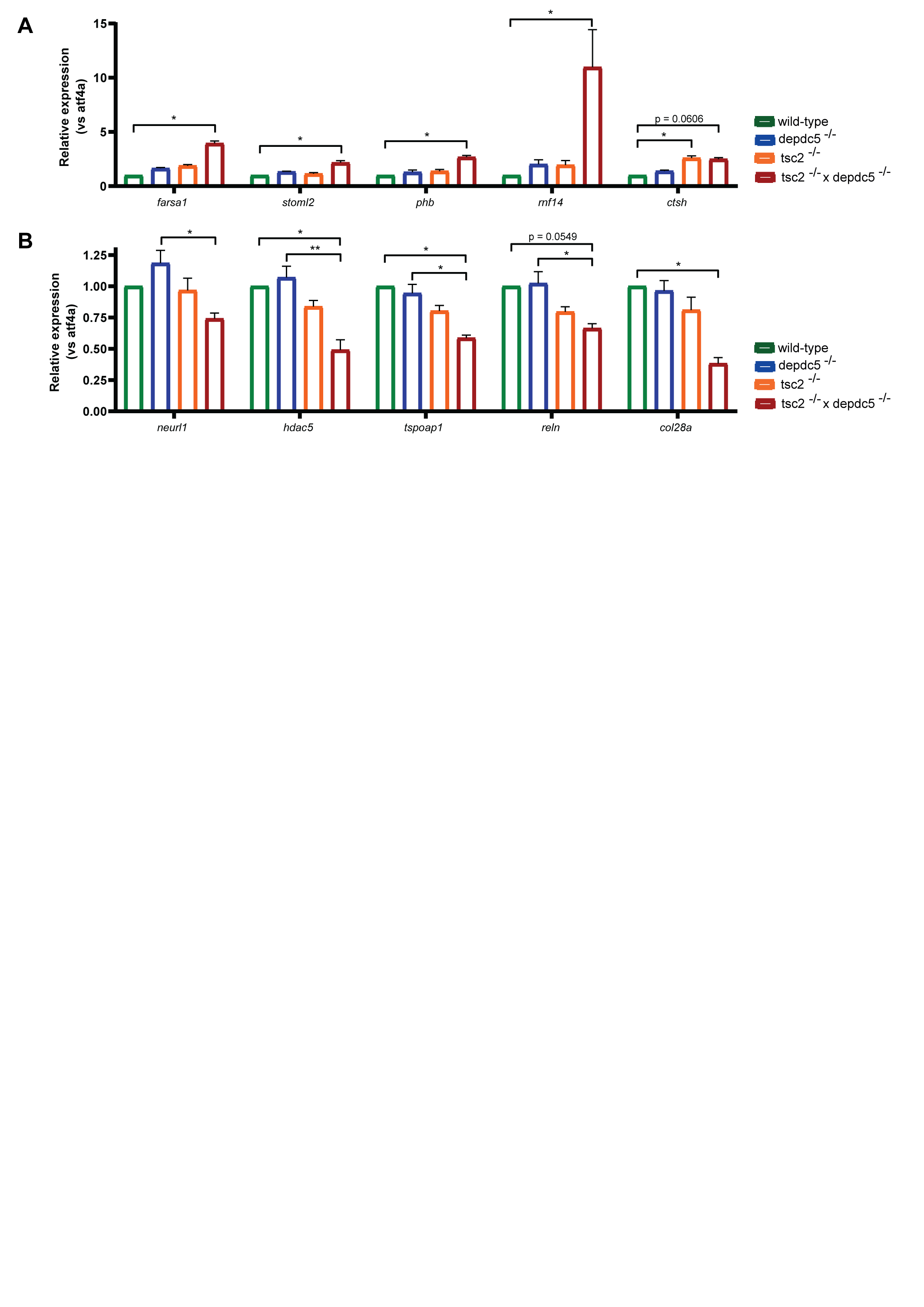

Supplement: Supplementary file 3 [file Image2.TIF]

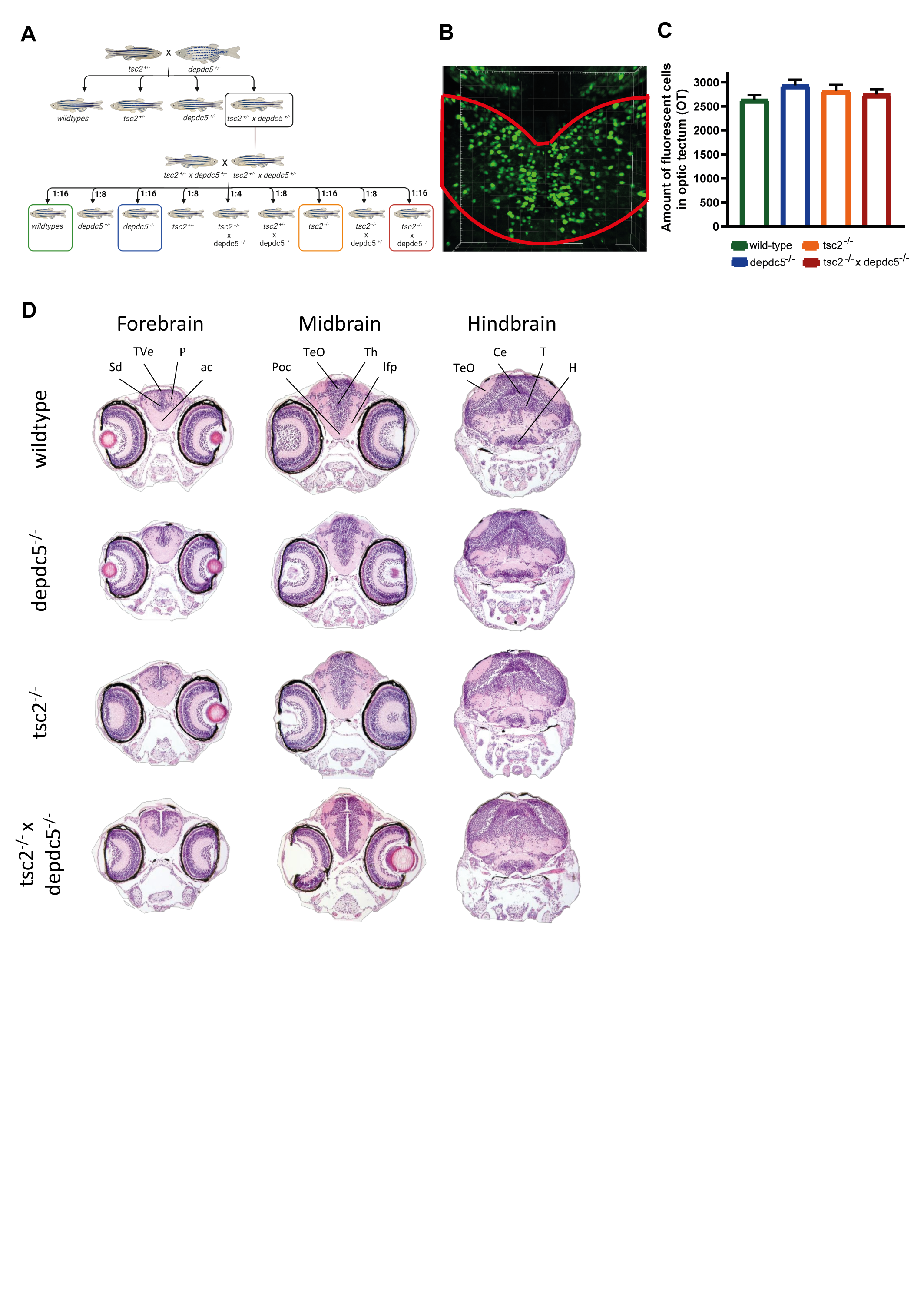

Supplement: Supplementary file 4 [file Image1.TIF]
