# Supplementary material for: Hyperactivation of mTORC1 in a double hit mutant zebrafish model of tuberous sclerosis complex causes increased seizure susceptibility and neurodevelopmental abnormalities
Source: Front Cell Dev Biol. 2022 Sep 27;10:952832. doi: 10.3389/fcell.2022.952832 (PMC9552079; doi:10.3389/fcell.2022.952832)
Supplement: Supplementary file 6 [file DataSheet1.pdf]

Supplementary Material

-- Double mutant zebrafish model of TSC --

1 Supplementary Data

1.1 Supplementary Tables

Supplementary Table 1: List of primers (written in 5′ to 3′ direction) used for RT-qPCR for validation of RNA sequencing.

Supplementary Table 2: Differential expressed gene (DEG) lists for *depdc5*$^{-/-}$, *tsc2*$^{-/-}$ and *depdc5*$^{-/-}$ x *tsc2*$^{-/-}$ 5 dpf zebrafish compared to wild-type larvae.

Supplementary Table 3: KEGG enrichment analysis for *depdc5*$^{-/-}$, *tsc2*$^{-/-}$ and *depdc5*$^{-/-}$ x *tsc2*$^{-/-}$ 5 dpf zebrafish compared to wild-type larvae and GO enrichment analysis for *tsc2*$^{-/-}$ and *depdc5*$^{-/-}$ x *tsc2*$^{-/-}$ 5 dpf zebrafish compared to wild-type larvae.

Supplementary Table 4: KEGG and GO enrichment analysis for *depdc5*$^{-/-}$ x *tsc2*$^{-/-}$ 5 dpf zebrafish compared to *tsc2*$^{-/-}$ larvae.

Supplementary Table 5: GO enrichment analysis on overlapping genes between *depdc5*$^{-/-}$ x *tsc2*$^{-/-}$ 5 dpf zebrafish larvae and human SEGA lesions.

Supplementary Table 6: Hypergeometric testing of mitochondrial genes in *tsc2*$^{-/-}$, *depdc5*$^{-/-}$ x *tsc2*$^{-/-}$, SEGA transcriptome profile and in the overlap between *tsc2*$^{-/-}$ and SEGA and overlap between *depdc5*$^{-/-}$ x *tsc2*$^{-/-}$ and SEGA transcriptomes.

Supplementary Table 7: Summary of results derived with the DGIdb database using the overlapping up-regulated and down-regulated DEGs between *depdc5*$^{-/-}$ x *tsc2*$^{-/-}$ and SEGA transcriptomes as input.

1.2 Supplementary Figures

Supplementary Figure 1:

(A) Mating scheme for the generation of the double mutants. Mendelian ratio for the nine genotypes resulting of the double *tsc2*$^{+/-}$ x *depdc5*$^{+/-}$ heterozygotes is presented in bold. Made with Biorender.

(B) Representative image of confocal image of the GABAergic and glutamatergic neuronal networks in the optic tectum of 5 dpf wild-type larvae. GABAergic and glutamatergic cells are visualised by green and red fluorescence, respectively. Data are presented as mean ± SEM, n=12-20/condition. Significant values (one-way ANOVA) are noted as “ns” p>0.05.

(C) Total number of GABAergic and glutamatergic cells in the optic tectum of 5dpf wild-type, *depdc5*$^{-/-}$, *tsc2*$^{-/-}$ and double homozygous larvae.

(D) Representative 20x images of a forebrain, midbrain and early hindbrain section in wild-type, *depdc5*$^{-/-}$, *tsc2*$^{-/-}$ and double homozygous larvae at 5 dpf, n=4 larvae/group. Ac = anterior commissure, Ce = cerebellum, lfb = lateral forebrain bundle, H = hypothalamus, P = pallium, Poc = postoptic commissure, Sd = dorsal part of subpallium, T = tegmentum, TeO = optic tectum, TVe = telencephalic ventricle

Supplementary Figure 2:

(A)-(B) RT-qPCR analysis for quantification of *farsa1, stoml2, phb, rnf14,* and *ctsh* **(A)** *neurl1, hdac5, tspoap1, reln* and *col28a1a* **(B)** mRNA levels *wild-type, depdc5*$^{-/-}$*, tsc2*$^{-/-}$*,* and double homozygous larvae. Data are presented as mean ± SEM, n=3-4/condition. Significant values (Kruskal-Wallis) are noted as *** $p \leq 0.001$, ** $p \leq 0.01$ and * $p \leq 0.05$
